# Balloon-Expandable Versus Self-Expandable Transcatheter Aortic Valve Implantation Devices: A Contemporary Systematic Review

**DOI:** 10.3390/jcm15187224

**Published:** 2026-09-17

**Authors:** Ioannis Apergis, Georgios P. Georghiou, Panos Georghiou, Efthalia Emmanouela Chrisofos, Filippos Triposkiadis, Konstantinos Lampropoulos

**Affiliations:** 1 School of Medicine, European University Cyprus, Nicosia 2404, Cyprus; japergis1@icloud.com (I.A.); thaliachrisofou@gmail.com (E.E.C.); ftriposkiadis@gmail.com (F.T.); lampropouloskon@gmail.com (K.L.); 2 Department of Surgery, Gray Faculty of Medical and Health Sciences, Tel Aviv University, Tel Aviv 6997801, Israel; 3 Barts and The London School of Medicine and Dentistry, Queen Mary University of London, Turner St., London E1 2AD, UK; panos.georghiou@gmail.com

**Keywords:** transcatheter aortic valve implantation, TAVI, transcatheter aortic valve replacement, TAVR, balloon expandable valve, BEV, self-expandable valve, SEV, aortic stenosis, systematic review

## Abstract

**Background/Objectives**: Transcatheter aortic valve implantation (TAVI) is an established treatment for severe aortic stenosis. Prosthesis selection is increasingly important because balloon-expandable valves (BEVs) and self-expandable valves (SEVs) differ in deployment, hemodynamics, and lifetime-management implications. This systematic review synthesized contemporary evidence comparing BEVs and SEVs, while platform-, generation-, and subgroup-specific studies provided contextual evidence. **Methods**: A PubMed-only systematic search was performed on 25 January 2026 for studies published between 1 January 2020 and 31 December 2025. Eligible human studies evaluated contemporary balloon or self-expandable systems. Randomized and observational studies, including single-arm cohorts, were included. Risk of bias was assessed using the revised Cochrane Risk of Bias tool (RoB 2) and the Methodological Index for Non-Randomized Studies (MINORS). Because of heterogeneity, findings were synthesized narratively without meta-analysis. Direct randomized and adjusted comparative evidence received greatest weight, whereas single-arm and within-platform studies were not interpreted as establishing comparative superiority. **Results**: Fifty studies met the eligibility criteria. No consistent mortality advantage was identified for either expansion mechanism. The most reproducible differences concerned hemodynamics, paravalvular regurgitation, and conduction outcomes. Supra-annular SEVs were more consistently associated with lower residual gradients and less prosthesis–patient mismatch, whereas BEVs showed lower paravalvular regurgitation or permanent pacemaker implantation in selected comparative settings. These signals varied by valve generation, implantation technique, anatomy, and study design. Evidence on coronary reaccess, durability, thrombosis, and reintervention was less extensive. **Conclusions**: Contemporary evidence does not support a universal BEV-versus-SEV hierarchy. Prosthesis selection should be individualized according to anatomy, conduction risk, hemodynamic priorities, and lifetime-management considerations. Comparative conclusions should remain generation-specific and distinguish direct comparative evidence from single-arm or anatomically selected cohorts. The review was not prospectively registered and received no external funding.

## 1. Introduction

### 1.1. Theoretical Background

Population-based echocardiographic data demonstrate that moderate or severe native valvular heart disease becomes increasingly prevalent with age, reaching 13.3% in adults aged 75 years and older [1]. In a meta-analysis of population-based studies, the prevalence of all aortic stenosis among elderly individuals was approximately 12.4%, while severe aortic stenosis occurred in approximately 3.4% [2]. In the same analysis, most elderly patients with severe aortic stenosis were symptomatic and a substantial proportion were not treated surgically, underscoring a large clinical and health-system burden in older populations [2].

Calcific aortic valve disease is the predominant etiology of aortic stenosis in contemporary high-income settings and is characterized by progressive valve mineralization and narrowing that imposes chronic left ventricular pressure overload [3]. Evidence indicates that calcific aortic valve disease is an active, multifactorial process involving both inflammatory and lipid-related pathways [3]. Despite advances in understanding pathobiology, no pharmacologic therapy has been shown to reliably halt or reverse progression of calcific aortic valve disease, making valve replacement the definitive therapy for severe disease [4]. Severe symptomatic aortic stenosis managed without aortic valve replacement is associated with a poor prognosis, with many patients dying within a few years after diagnosis [5].

In a United States academic cohort, nonadherence to guideline-indicated aortic valve replacement was common and was associated with reasons such as perceived surgical risk, comorbidity burden, and referral patterns [6]. A large contemporary real-world analysis further suggested substantial mortality risk across the spectrum of untreated aortic stenosis severity and low rates of valve replacement among patients with severe disease [7]. Current American College of Cardiology/American Heart Association guidance emphasizes individualized selection of the mode of aortic valve intervention according to operative risk, anatomy, comorbidity, life expectancy, and patient preferences [8]. The 2025 European Society of Cardiology/European Association for Cardio-Thoracic Surgery (ESC/EACTS) Guidelines further refine this approach by integrating age, anatomical suitability, anticipated longevity, and lifetime-management considerations into Heart Team decision-making [9]. In anatomically suitable patients aged ≥ 70 years with tricuspid aortic valve stenosis and feasible transfemoral access, transcatheter aortic valve implantation (TAVI) is recommended as the primary treatment modality, whereas bicuspid aortic stenosis is considered separately because of its distinct morphology, frequent associated aortopathy, asymmetric calcification, and more limited randomized TAVI evidence [9]. Consequently, findings derived predominantly from tricuspid aortic stenosis populations should not be assumed to apply directly to bicuspid anatomy.

TAVI, also referred to as transcatheter aortic valve replacement (TAVR), demonstrated superiority over standard medical therapy in patients considered inoperable for surgery in the PARTNER trial [10]. In high-risk surgical candidates, transcatheter valve re-placement using an early-generation balloon-expandable system was shown to be noninferior to surgical aortic valve replacement with respect to all-cause mortality at one year [11]. Randomized evidence also supported self-expanding transcatheter valve re-placement in high-risk patients, with outcomes favorable compared with surgery [12]. In intermediate-risk patients, the PARTNER 2 trial reported similar rates of death or disabling stroke for transcatheter versus surgical replacement at two years [13]. In intermediate-risk patients, the SURTAVI trial showed noninferiority of self-expanding TAVI compared with surgery for the composite of death from any cause or disabling stroke at 24 months [14]. In low-surgical-risk patients, the PARTNER 3 trial reported favorable one-year composite outcomes for contemporary balloon-expandable transfemoral TAVI compared with surgery [15]. In low-risk patients, self-expanding supra-annular TAVI was noninferior to surgery for death or disabling stroke at 24 months in the Evolut Low Risk trial [16].

### 1.2. Existing Knowledge

As TAVI expands toward younger and lower-risk populations, valve selection has become increasingly important because device choice may influence both immediate procedural safety and longer-term lifetime management considerations [17]. Balloon-expandable valves (BEVs) and self-expandable valves (SEVs) have distinct design features, implantation characteristics, and hemodynamic profiles, and available evidence suggests that each platform can confer context-dependent advantages and limitations [17]. A propensity-matched analysis from a national registry suggested higher paravalvular regurgitation and higher in-hospital and two-year mortality with self-expanding compared with balloon-expandable transcatheter valves, reinforcing the need for careful comparative evaluation [18]. Randomized head-to-head comparisons have also reported device-specific trade-offs, including differences in device success and early clinical outcomes when balloon-expandable and self-expanding systems were directly compared in the CHOICE trial [17]. The SCOPE I trial found that a self-expanding ACURATE neo system did not meet noninferiority compared with the balloon-expandable SAPIEN 3 device based on an early composite safety and efficacy endpoint [19]. The SCOPE 2 trial compared ACURATE neo with CoreValve Evolut and reported differences in outcomes including permanent pacemaker implantation at 30 days [20]. In anatomically challenging patients with a small aortic annulus, the randomized SMART trial showed noninferior clinical outcomes with a self-expanding supra-annular valve versus a balloon-expandable valve and differences in bioprosthetic-valve dysfunction through 12 months [21]. Standardized endpoint definitions are essential for interpreting heterogeneous device studies, and the Valve Academic Research Consortium 3 document provides updated consensus endpoint definitions for transcatheter and surgical aortic valve clinical research [22]. Conduction disturbances and permanent pacemaker implantation remain frequent and clinically consequential issues after TAVI, with implications for device selection, implantation technique, and follow-up pathways [23]. Meta-analytic evidence suggests that permanent pacemaker implantation after TAVI is associated with higher long-term risks of all-cause mortality and heart-failure rehospitalization [24]. Paravalvular leak is clinically important because meta-analysis of Kaplan–Meier-derived individual patient data associated even mild paravalvular re-gurgitation after TAVI with higher risks of adverse outcomes [25]. Coronary access after TAVI can be challenging in a substantial proportion of patients, and prosthesis design characteristics may influence feasibility of future coronary angiography or intervention [26]. Commissural alignment has been associated with improved rates of selective coronary access after TAVI with supra-annular valves, although risk of unfeasible or non-selective access persists in some patients [27]. Meta-analysis of available randomized trials emphasizes that broad platform-level differences between balloon-expandable and self-expanding valves can be nuanced and endpoint-dependent while also highlighting that comparisons are limited by the number of randomized trials and the evolution of device generations [28].

A further consideration in the contemporary TAVI literature is that “platform” comparison involves not only clinical outcomes but also distinct structural solutions to restoring forward flow across a calcified and anatomically variable aortic root. Interpretation should therefore account for frame design, leaflet position, sealing mechanisms, delivery systems, and device generation, because these characteristics may influence hemodynamics, paravalvular regurgitation (PVL), conduction-system interaction, implantation control, and future coronary access. Within the balloon-expandable category, the SAPIEN 3/Ultra/Ultra RESILIA family retains a relatively low-frame, balloon-deployed architecture with bovine pericardial leaflets, while later iterations incorporated a taller external sealing skirt to improve sealing. This configuration may facilitate controlled implantation and make coronary engagement less dependent on ideal commissural alignment than with taller supra-annular systems, although reaccess can still be challenging in a narrow sinotubular junction or after redo-TAVI [17,29,30]. By contrast, the CoreValve/Evolut family uses a tall, supra-annular self-expanding frame. Its leaflet position and geometry are associated with lower residual gradients and larger effective orifice area, particularly in smaller annuli, but may also increase interaction with the left ventricular outflow tract, conduction system, and coronary reaccess pathways [17,29,30]. Evolut FX studies illustrate how refinements in orientation, implant depth, and commissural alignment address limitations of earlier generations [31,32].

Generational refinement is likewise evident in the ACURATE, Portico, and Navitor platforms. ACURATE neo and neo2 are supra-annular nitinol self-expanding valves with stabilization arches and porcine pericardial leaflets; neo2 incorporated an approximately 60% enlarged outer sealing skirt to improve adaptation to irregular calcified anatomy and reduce PVL [33,34]. Limited protrusion of the lower crown into the left ventricular outflow tract (LVOT) was also intended to reduce conduction interaction [34]. Within the Abbott family, Portico is a self-expanding intra-annular valve with bovine pericardial leaflets in a nonflared nitinol frame and a pericardial sealing cuff. The FlexNav delivery system introduced a lower-profile hydrophilic sheath and stability layer intended to reduce vascular trauma and improve deployment precision [35]. Navitor is similarly repositionable and self-expanding but incorporates a non-tapered frame, large stent cells, intra-annular bovine pericardial leaflets, and an outer NaviSeal cuff designed to reduce PVL while preserving coronary access [36,37]. These examples illustrate why successive generations should not be obscured by broad BEV-versus-SEV classification alone.

The balloon-expandable Myval family represents a different approach centered on anatomical matching. Myval uses a hybrid honeycomb nickel–cobalt frame with bovine pericardial leaflets and a broad size matrix including intermediate and extra-large options; the LANDMARK trial also included the Myval Octacor variant [38,39]. This sizing range aims to reduce undersizing or oversizing in borderline annuli, which may influence PVL, prosthesis–patient mismatch, and conduction injury. The Hydra transcatheter heart valve is a self-expanding supra-annular system with bovine pericardial leaflets, a high sealing skirt, large open mid-frame cells for coronary access, and a nonflared inflow intended to reduce conduction interference. Its segmented radial-force profile, recapturability, and flexible delivery catheter reflect efforts to balance anchoring, hemodynamics, and deliverability in challenging anatomy [40].

Several less frequently represented platforms further illustrate this evolution, although their clinical evidence base is more limited. The Vienna Aortic Valve Self-Expandable System is a fully premounted, supra-annular nitinol valve with bovine pericardial leaflets, an outer sealing skirt, optional complete recapture, dedicated commissural markers, and relatively large stent cells intended to facilitate coronary reaccess [41]. ALLEGRA is a self-expanding supra-annular valve with bovine leaflets whose supra-annular position is intended to preserve effective orifice area and low gradients, consistent with favorable short-term hemodynamics reported in its multicenter registry [42]. JenaValve differs structurally by using locator-guided positioning and leaflet-clipping fixation, enabling anchoring to native cusps even when substantial calcification is absent and thereby supporting treatment of pure aortic regurgitation [43,44].

VitaFlow and VitaFlow Liberty were developed with complex calcified and bicuspid anatomy in mind. VitaFlow combines bovine pericardial leaflets with a double-layer sealing skirt, whereas VitaFlow Liberty adds a hybrid-density stent and retrievable motorized delivery system to improve flexibility, positioning, and deployment control [45,46]. Finally, the balloon-expandable Inovare Proseal has been evaluated by post-implantation computed tomography (CT), with reported preservation of frame circularity and expansibility, favorable residual gradients, low rates of significant PVL, and maintained coronary ostial relationships [47]. Although evidence for these less represented systems remains less extensive than for SAPIEN or Evolut, their structural concepts remain relevant because contemporary valve development increasingly targets specific challenges such as absent calcium, bicuspid morphology, calcific burden, sealing, conduction preservation, delivery control, and future coronary access. Taken together, these platform- and generation-specific characteristics provide mechanistic context for the clinical findings summarized in this review; however, design features alone should not be interpreted as evidence of comparative clinical superiority.

### 1.3. Description of the Problem

Despite this growing literature over the past 5 years, the comparative evidence base remains difficult to translate into clear TAVI platform-level recommendations, since the studies that exist differ with respect to patient risk profile, access route, device generation, endpoint definitions, and duration of follow-up [22,28]. Moreover, clinically relevant outcomes (such as permanent pacemaker implantation, paravalvular regurgitation, hemodynamic performance, and future coronary access) may not favor the same platform across all anatomical and clinical scenarios [23,25,26]. Although randomized head-to-head comparisons are increasingly available, the number of trials remains limited, and the rapid evolution of valve technology continues to complicate cross-study interpretation [28]. Therefore, a focused systematic review is needed to synthesize contemporary evidence, clarify the principal trade-offs between balloon-expandable and self-expanding platforms, and identify areas in which uncertainty persists to exist.

### 1.4. Purpose and Specific Objectives

The primary objective of this systematic review is to synthesize and critically appraise contemporary evidence comparing balloon-expandable and self-expanding transcatheter aortic valves in adults undergoing TAVI for severe symptomatic aortic stenosis. The review focuses on the principal valve families represented in the contemporary literature, including Edwards SAPIEN, Medtronic CoreValve/Evolut, Boston Scientific ACURATE neo/neo2, Abbott Navitor and Portico/FlexNav, Meril Myval/Myval Octacor, Hydra, ALLEGRA, and the Vienna self-expandable system, while also recording underrepresented platforms such as JenaValve, VitaFlow/VitaFlow Liberty, and Inovare when identifiable. Direct comparative evidence forms the principal basis for BEV-versus-SEV interpretation, whereas platform-, generation-, procedural-, and subgroup-specific studies provide contextual evidence and are not considered capable of establishing comparative superiority when a direct comparator is absent. Specifically, the objectives are [a] to compare major clinical outcomes across valve platforms, [b] to evaluate device-related complications and valve performance, [c] to assess procedural and anatomical factors that may influence lifetime management, including future coronary access, and [d] to identify persisting evidence gaps relevant to future research and clinical decision-making.

### 1.5. Enhancement of Existing Knowledge—Added Value and Benefit

This review addresses these objectives within the contemporary literature at a time when valve-platform selection has become increasingly consequential as TAVI is offered to younger and lower-risk patients, particularly those who may later require coronary access or repeat valve intervention [17,26,27]. Its relevance also extends beyond the index procedure. As TAVI volumes continue to grow and treatment is applied to patients with longer life expectancy, the absolute number of device failures, reinterventions, and explantations will also increase. In that setting, a clear understanding of valve-specific design characteristics, modes of failure, and reintervention constraints becomes important not only at the time of initial implantation, but also when later decisions about coronary procedures, redo-TAVI, or surgical explantation must be made. By assembling evidence from randomized and observational studies and emphasizing clinically meaningful trade-offs rather than isolated endpoints, this review may support more individualized Heart Team decision-making and better align prosthesis choice with anatomy, comorbidity profile, and lifetime management strategy [9]. It may also help distinguish findings that appear consistent across contemporary studies from those that remain uncertain because direct comparative evidence is still limited and device technology continues to evolve [28]. In this respect, the review may be relevant both to current clinical practice and to the shaping of future research priorities in structural heart disease.

## 2. Materials and Methods

### 2.1. Review Design and Scope

The conduction of this systematic review was performed in accordance with the Preferred Reporting Items for Systematic Reviews and Meta-Analyses (PRISMA) 2020 statement [48] and it was designed as a systematic review without meta-analysis. The aim was to identify and synthesize contemporary evidence on balloon-expandable and self-expanding transcatheter aortic valve replacement/implantation (TAVR/TAVI) systems across prespecified clinical, procedural, and anatomical domains. Because several reports described broader device families rather than individual device generations, evidence was classified at model level whenever possible and otherwise at valve-family level.

### 2.2. Registration and Protocol

This systematic review was not registered in a public systematic-review registry. A written protocol was developed before commencement of the literature search as part of the first author’s Doctor of Medicine (M.D.) thesis at European University Cyprus. Because registration had not been completed before study selection and data extraction, the review was not subsequently registered retrospectively. The protocol is available from the corresponding author upon reasonable request. Deviations from the original protocol are summarized in Supplementary Table S3.

### 2.3. Description of Search Strategy

A PubMed-only search, prespecified in the thesis protocol, was performed on 25 January 2026 and covered publications from 1 January 2020 through 31 December 2025. The strategy combined three main components: (a) device-platform terms for balloon-expandable and self-expanding transcatheter heart valves, (b) TAVR/TAVI procedure terms, and (c) prespecified clinical and anatomical outcome terms. The final strategy additionally excluded studies focused on non-target access routes and review-level publications and applied limits for human studies, original clinical studies, and publication date. The stepwise search history and representative keywords are summarized in Table 1.

#### Exact Final PubMed Query

The query reproduces the final PubMed history (10) exactly as used for the revised search on 25 January 2026 and it is provided as Table S1 in the Supplementary Materials list.

### 2.4. Eligibility Criteria

Eligible studies were human studies indexed in PubMed that evaluated contemporary balloon-expandable or self-expanding TAVR/TAVI systems prespecified in the protocol framework. Included designs comprised randomized controlled trials; comparative observational studies, including cohort, case–control, and registry-based analyses; prospective or retrospective cohort studies; post hoc analyses with extractable data; and feasibility or first-in-human studies. Studies were excluded if they were case reports, editorials or letters without primary data, reviews or protocol-only papers, animal or preclinical studies, surgical aortic valve replacement (SAVR)-only studies, or reports focused on mechanically expandable valves or mixed-platform datasets without extractable balloon-expandable versus self-expanding data. Such devices were recorded for contextual evidence mapping only and were not treated as eligible comparator platforms or as a basis for the review’s platform-level conclusions. The review further excluded non-target access routes and publications outside the predefined 2020–2025 window. Consistent with the operational thesis workflow, only freely accessible full-text reports available at the time of screening were advanced to the working screening dataset.

### 2.5. Study Selection Process

**Figure 1:**
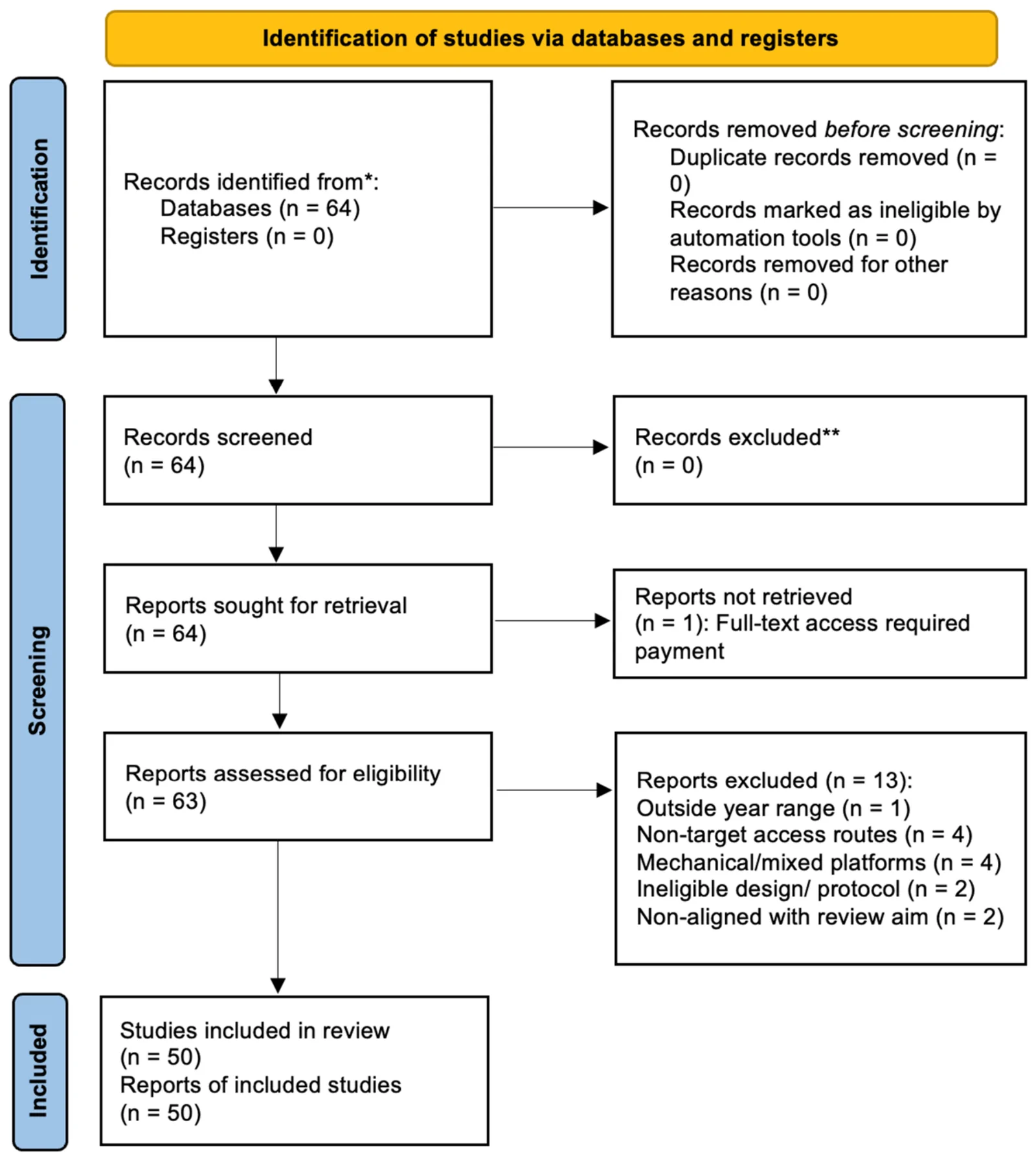
Preferred Reporting Items for Systematic Reviews and Meta-Analyses (PRISMA) 2020 flow diagram of study identification, screening, eligibility assessment, and inclusion. * Records were identified exclusively through PubMed; no additional registers were searched. ** No records were excluded dring title/abstract screening, and no automation tools were used.

Records retrieved by the final search were screened in two sequential stages. The first stage was comprised by title/abstract review by I.A. The second stage followed with the final full-text eligibility assessment I.A. and independently verified by G.P.G. Eligibility uncertainties were resolved through discussion and, when necessary, with K.L. No duplicate records were identified within the PubMed set, and no automation tools or additional registers were used. Decisions were made against the prespecified eligibility criteria, and reasoning for exclusion was documented at full-text review. The detailed numerical flow of records through both screening and eligibility assessment is presented in Results Section 3.1 and illustrated in Figure 1.

### 2.6. Data Extraction

Data were extracted in a structured evidence-mapping format aligned with the review objectives. Data were extracted by I.A. using a standardized evidence-extraction form and were subsequently verified by G.P.G. for accuracy, completeness, and consistency. Discrepancies or uncertainties were resolved through discussion. Study authors were not contacted for missing information, and unclear or unavailable data were recorded as not reported. For each included study, extracted variables comprised author and year, geographic setting, study design, center type, sample size, valve category, named valve family or model, comparison structure, target population or anatomical subgroup, follow-up duration, and the main clinical, procedural, imaging, or hemodynamic outcomes relevant to the prespecified domains. Outcomes and clinical, procedural, and anatomical domains of interest included all-cause mortality, stroke, permanent pacemaker implantation and conduction disturbances, thrombocytopenia or platelet-count reduction; acute kidney injury; paravalvular leak, transvalvular hemodynamic performance, including mean pressure gradients, effective orifice area, and prosthesis–patient mismatch, bicuspid aortic valve anatomy, horizontal aorta or aortic angulation, small aortic annulus, infective endocarditis, coronary access and coronary obstruction, commissural alignment, native cusp calcification, left ventricular systolic dysfunction, sex-specific differences, balloon aortic valvuloplasty before TAVI, other procedural complications, and valve reintervention. All relevant reported follow-up time points were extracted when available.

### 2.7. Quality Assessment

Methodological quality was appraised using design-appropriate tools. Risk-of-bias assessments were initially performed by I.A. and independently verified by G.P.G. Disagreements were resolved by consensus, with consultation of K.L. when necessary. Randomized controlled trials were evaluated with the revised Cochrane Risk of Bias tool (RoB 2), whereas nonrandomized studies were assessed with the Methodological Index for Non-Randomized Studies (MINORS), using the comparative or noncomparative version as appropriate. Post hoc analyses were appraised according to the design of the parent study, with additional attention to the transparency and prespecification of the secondary analysis. Quality assessment informed interpretation of the findings but was not used as an independent exclusion criterion once the core eligibility requirements had been met.

### 2.8. Effect Measures

Binary outcomes were reported as event numbers and percentages and, where provided by the original studies, as risk ratios, odds ratios, or hazard ratios with 95% confidence intervals. Continuous outcomes were reported as means with standard deviations or medians with interquartile ranges, according to the original study reporting. No effect estimates were recalculated or statistically pooled.

### 2.9. Data Synthesis

Studies were synthesized according to both evidence type and prespecified clinical or anatomical domain. For comparative interpretation, evidence was considered hierarchically. Direct randomized BEV-versus-SEV comparisons were given the greatest interpretative weight, followed by adjusted or propensity-matched comparative observational studies and then unadjusted comparative cohorts. Within-platform or generation-specific comparisons and single-arm studies were used primarily to characterize device-specific performance, safety, technological evolution, and evidence gaps and were not interpreted as establishing comparative superiority between BEVs and SEVs. Findings from non-randomized studies were interpreted as associations rather than causal effects because statistical adjustment and propensity-based methods cannot eliminate residual or unmeasured confounding. Study characteristics and individual findings were summarized in tables and synthesized narratively, with emphasis on recurrent outcome patterns, consistency across study designs, and areas of uncertainty. Meta-analysis, meta-regression, statistical subgroup analyses, and sensitivity analyses were not undertaken because of substantial clinical and methodological heterogeneity in study design, patient populations, valve platforms and generations, outcome definitions, follow-up durations, and completeness of reporting. Missing summary statistics were not imputed, and no data conversions were performed.

### 2.10. Assessment of Reporting Bias

Formal assessment of reporting bias, including funnel-plot analysis, was not performed because no meta-analysis was undertaken and insufficient numbers of directly comparable studies were available within individual outcome domains. Potential publication, retrieval, and selective-reporting biases were considered qualitatively during interpretation.

### 2.11. Certainty of Evidence

A formal Grading of Recommendations Assessment, Development and Evaluation (GRADE) assessment was not prespecified and was not performed. To provide a structured appraisal, major outcome domains were qualitatively assessed according to study design, risk of bias, consistency, directness, and breadth and duration of follow-up. Evidence strength was categorized as stronger, moderate, or limited within the context of this review. These categories are intended to aid interpretation and should not be considered equivalent to formal GRADE certainty ratings.

## 3. Results

### 3.1. Study Selection

The final PubMed search yielded 64 records. No records were excluded at the screening stage. Full texts were sought for 64 reports; 1 report could not be retrieved because access required payment. Accordingly, 63 reports were assessed in full for eligibility. After full-text review, 13 reports were excluded: 1 was outside the predefined publication window, 4 involved non-target access routes, 4 evaluated mechanical or mixed platforms without extractable contemporary balloon-expandable versus self-expanding data, 2 were ineligible by design or were protocol-only reports, and 2 were not aligned with the review aim. The final qualitative synthesis included 50 studies. The process of study selection is summarized in Table 2 and illustrated in Figure 1.

### 3.2. Evidence Structure and Methodological Quality

The final evidence base comprised 50 studies published between 2020 and 2025 and was characterized by substantial methodological heterogeneity. Four publications were derived from randomized evidence, whereas the remaining 46 were non-randomized and included adjusted or propensity-matched comparative analyses, multicentre and single-centre registries, prospective and retrospective cohorts, post hoc analyses, and single-arm or feasibility studies. Direct BEV-versus-SEV comparisons therefore represented only part of the overall evidence base; many studies instead evaluated individual valve platforms or generations, procedural strategies, or selected anatomical and clinical subgroups. Follow-up ranged from procedural or predischarge assessment to 5 years, although short-term and 1-year outcomes predominated. Detailed study characteristics are provided in Supplementary Table S2.

The four randomized-trial-derived publications were assessed using RoB 2. None was judged to be at high overall risk of bias for the critical outcomes assessed, although all raised some concerns. These were principally related to open-label study design, secondary or subgroup analyses, and incomplete longer-term follow-up. Subgroup analyses in which the population of interest had not been independently randomized were considered particularly vulnerable to imbalance and selective interpretation, while incomplete long-term imaging follow-up reduced confidence in some longitudinal valve-performance estimates. Conversely, randomized allocation, predefined analytical frameworks, central clinical-event adjudication, and standardized imaging assessment, where available, strengthened the reliability of these studies and gave them greater interpretative weight in the comparative synthesis.

Methodological quality among the 46 non-randomized studies, assessed using MINORS, varied substantially. The more informative observational evidence generally came from larger multicentre comparative analyses incorporating propensity-score matching, inverse-probability weighting, or multivariable adjustment. Such methods improved comparability between treatment groups but could not eliminate residual or unmeasured confounding. Other studies were predominantly prospective or retrospective single-arm cohorts, device-specific registries, small single-centre series, and early-feasibility investigations. Common limitations included absence of a concurrent comparator, selected patient populations, retrospective data collection, relatively short follow-up, variable endpoint definitions and ascertainment methods, and incomplete adjustment for baseline differences.

Accordingly, randomized and well-adjusted comparative studies formed the principal basis for BEV-versus-SEV interpretation. Single-arm, within-platform, generation-specific, and early-feasibility studies remained clinically informative for characterizing device-specific safety, performance, technological evolution, and uncommon anatomical scenarios, but were not interpreted as demonstrating comparative superiority. Findings from non-randomized studies were therefore treated as associations rather than causal effects. Detailed RoB 2 and MINORS assessments are provided in Supplementary Tables S4 and S5.

### 3.3. Direct Comparative BEV-Versus-SEV Evidence

Direct comparative evidence demonstrated clinically relevant trade-offs rather than consistent superiority of either expansion mechanism. The strongest evidence came from randomized comparisons. In the LANDMARK trial-derived analysis, 768 patients were randomized to Myval, SAPIEN, or Evolut. Myval was non-inferior to both comparator families for the 30-day primary composite endpoint, while permanent pacemaker implantation was similar across groups. Hemodynamic performance differed across platforms: Myval showed more favorable hemodynamics than SAPIEN, whereas Evolut showed more favorable hemodynamics than Myval; moderate-to-severe prosthetic regurgitation was numerically more frequent with Evolut than with Myval. These findings illustrate that similar early clinical performance may coexist with differences in valve-specific hemodynamic and regurgitation profiles [39].

Longer-term randomized evidence was provided by the Evolut R versus SAPIEN 3 comparison. Among 447 randomized patients, the predefined valve-related composite endpoint at 5 years was similar between SEVs and BEVs (67.7% vs. 63.4%; hazard ratio (HR) 0.89, 95% confidence interval (CI) 0.70–1.13; *p* = 0.34). SEV was associated with a lower stroke rate in this comparison and with lower mean transvalvular gradients among patients with available 5-year echocardiographic follow-up (7 vs. 11 mmHg). The incomplete long-term imaging dataset warrants caution in interpreting the hemodynamic comparison, but the study provides important randomized evidence that lower gradients with a self-expanding valve did not translate into superiority for the principal composite clinical outcome [49].

Adjusted observational comparisons broadly reinforced the presence of endpoint-specific trade-offs. In the long-term propensity-matched analysis by Bihag et al., 683 BEV recipients and 680 SEV recipients had similar in-hospital mortality and stroke, 1-year mortality and readmission, and late all-cause mortality over a median follow-up of approximately 77 months. BEV was associated with lower permanent pacemaker implantation (8.3% vs. 13.4%),whereas SEV was associated with lower gradients, larger effective orifice area, and less prosthesis-patient mismatch (PPM). Differences in subclinical leaflet thrombosis and transient ischemic attack were also observed, emphasizing that comparative profiles extended beyond the conventional gradient–PVL–pacemaker triad [50].

The direction and clinical importance of these differences varied in anatomically or clinically selected populations. In patients with left ventricular systolic dysfunction, a propensity-matched analysis of 134 BEV–SEV pairs found lower gradients but more residual paravalvular regurgitation with SEV; adjusted 5-year all-cause mortality was also higher with SEV, although this association arose from a single observational subgroup and residual confounding cannot be excluded [51]. In the TAVI-SMALL 2 registry of 1378 patients with small annuli, supra-annular/self-expanding valves were associated with lower gradients and less severe prosthesis–patient mismatch but more than mild PVL than intra-annular/balloon-expandable valves, without a significant difference in all-cause mortality [52]. Conversely, in large and extra-large annuli, SAPIEN 3 was associated with higher overall device success and less moderate-to-severe aortic regurgitation than Evolut R, while mortality was similar; these differences were attenuated in the extra-large-annulus subgroup [53].

Smaller contemporary comparative studies provide additional context but should receive less interpretative weight. In the propensity-matched Myval-versus-Portico comparison, short- and mid-term mortality, pacemaker implantation, and device failure were similar, whereas adjunctive ballooning was more frequently required with Portico [38]. Taken together, the direct comparative evidence does not support a universal BEV-versus-SEV hierarchy. Instead, it consistently indicates trade-offs whose magnitude depends on the specific valve generation, anatomical setting, clinical phenotype, and outcome considered. Randomized findings were therefore given greatest interpretative weight, while adjusted observational comparisons were considered supportive and subgroup-specific associations were not interpreted as causal.

### 3.4. Outcome-Specific Synthesis of Clinical and Valve-Performance Findings

#### 3.4.1. Mortality, Stroke, and Rehospitalization

Across direct comparative studies, no reproducible class-level mortality advantage was identified for either BEV or SEV. Long-term propensity-matched contemporary BEV-versus-SEV data showed similar in-hospital, 1-year, and late mortality, together with similar 1-year readmission [50]. Randomized 5-year evidence likewise showed no significant difference in the principal valve-related composite outcome, although a lower stroke rate was observed with SEV in that specific Evolut R versus SAPIEN 3 comparison [49]. Conversely, the higher adjusted 5-year mortality observed with SEV in the reduced-left ventricular ejection fraction (LVEF) cohort was derived from a single observational propensity-matched analysis [51]. Mortality, stroke, and rehospitalization data therefore do not establish consistent expansion-mechanism superiority, and subgroup-specific associations should not be interpreted causally.

#### 3.4.2. Permanent Pacemaker Implantation and Conduction Disturbances

Conduction outcomes were among the most consistently reported domains. Several comparative datasets showed a higher pacemaker burden with self-expanding platforms, including the long-term matched SAPIEN-versus-Evolut analysis, in which permanent pacemaker implantation was less frequent with BEV [50]. However, pacing risk varied substantially according to valve generation and implantation technique. Contemporary Evolut FX studies incorporating refined orientation and cusp-overlap deployment reported lower pacemaker rates than earlier self-expanding experiences [31,32], while SAPIEN-specific analyses identified implantation depth, oversizing, valve size, and calcification as relevant predictors of conduction injury [54,55]. The available evidence therefore supports consideration of both device design and implantation strategy rather than attribution of conduction risk to expansion mechanism alone.

#### 3.4.3. Paravalvular Regurgitation

Comparative evidence generally favored balloon-expandable platforms with respect to residual paravalvular regurgitation, although the magnitude of this difference varied by generation and anatomy. In small annuli, self-expanding platforms were associated with more favorable hemodynamics but more than mild PVL [52], and a similar gradient–regurgitation trade-off was observed in the reduced-LVEF cohort [51]. Importantly, within-platform evolution altered this profile: ACURATE neo2 was associated with substantially less moderate-or-greater PVL than the preceding ACURATE neo generation [33], while contemporary Navitor studies reported low clinically relevant PVL in single-arm cohorts [36,37]. The latter findings demonstrate generation-specific improvement but cannot establish comparative superiority.

#### 3.4.4. Hemodynamics, Effective Orifice Area, and Prosthesis–Patient Mismatch

The most consistent self-expanding signal concerned hemodynamic performance, particularly for supra-annular platforms. Lower residual gradients, larger effective orifice areas, or less PPM were reported in randomized and adjusted comparative studies, including long-term contemporary BEV-versus-SEV comparisons and small-annulus cohorts [49,50,52,56]. Nevertheless, better resting hemodynamics did not consistently translate into improved hard clinical outcomes. In a 5-year SAPIEN 3 analysis of small annuli, neither early mean gradient nor PPM was associated with worse long-term outcomes within that cohort [57]. Hemodynamic differences should therefore be considered clinically relevant, particularly where residual gradient burden is expected to matter, but should not be interpreted as a surrogate for survival superiority.

#### 3.4.5. Coronary Access and Commissural Alignment

Evidence concerning coronary reaccess and commissural alignment was less extensive and was derived predominantly from CT-, angiography-, and procedure-focused studies rather than randomized clinical outcomes. Low-frame balloon-expandable systems and intentional orientation strategies for self-expanding platforms demonstrated different interactions with the coronary ostia and sinotubular junction [29,30]. In RE-ACCESS 2, systematic commissural alignment improved procedural orientation but did not completely eliminate unsuccessful coronary cannulation, which remained concentrated in Evolut recipients [58]. These findings are increasingly relevant to lifetime management but remain supported primarily by mechanistic and procedural rather than hard clinical evidence.

#### 3.4.6. Valve Thrombosis, Durability, and Reintervention

Long-term comparative evidence for valve thrombosis, structural valve deterioration, and reintervention remained considerably less developed than the evidence for early hemodynamics, PVL, or pacing. Selected contemporary cohorts reported low reintervention rates and generally infrequent clinically recognized valve thrombosis, but follow-up duration and adjudication were uneven across valve families [50,57,59,60]. No robust evidence established superior structural durability for either BEV or SEV. Consequently, current lifetime-management decisions should incorporate anticipated future coronary access, redo-TAVI feasibility, neo-skirt configuration, and potential surgical explantation rather than assuming that early hemodynamic differences predict superior long-term durability.

### 3.5. Clinically Relevant Anatomical and Functional Subgroups

#### 3.5.1. Bicuspid Aortic Valve

Bicuspid aortic stenosis was analyzed separately from conventional tricuspid disease because its morphology, calcification distribution, and associated aortopathy may alter procedural behavior and because randomized BEV-versus-SEV evidence remains limited. Contemporary Evolut studies demonstrated favorable procedural and 1-year outcomes in carefully selected low-risk bicuspid patients [61,62], while the ACURATE neo2 bicuspid registry provided additional device-specific feasibility data [63]. However, these studies do not constitute randomized BEV-versus-SEV comparisons. Accordingly, the present evidence supports the feasibility of selected platforms in appropriately screened bicuspid anatomy but does not establish class-level superiority.

#### 3.5.2. Small and Large Annuli

Annular size modified the relative importance of hemodynamics and sealing. In small annuli, supra-annular/self-expanding systems were generally associated with lower gradients and less PPM than intra-annular/balloon-expandable systems, although this advantage was accompanied by more residual PVL in selected comparisons [52,56]. Long-term SAPIEN 3 data suggested that higher early gradients or PPM within a small-annulus BEV population were not necessarily associated with worse 5-year outcomes [57]. Evidence in large or extra-large annuli was predominantly observational and included both BEV and SEV platforms [53,64]; these data support platform-specific feasibility but remain insufficient for a universal class preference.

#### 3.5.3. Reduced LVEF

Evidence in reduced LVEF was particularly limited. One propensity-matched analysis reported lower residual gradients with SEV but more residual regurgitation and a higher adjusted 5-year mortality signal than with BEV [51]. Because this finding arose from a single observational registry analysis, it should be considered hypothesis-generating rather than definitive evidence favoring one platform.

#### 3.5.4. Horizontal Aorta and Challenging Coronary Anatomy

Evidence for markedly horizontal aorta was largely device-specific, with Evolut implantation shown to be feasible using dedicated procedural strategies in highly selected patients [65]. Challenging coronary anatomy is better represented by coronary-access and commissural-alignment studies [29,30,58]. These phenotypes should therefore influence procedural planning and device-generation selection but currently lack sufficiently broad comparative evidence to support rigid BEV-versus-SEV recommendations.

#### 3.5.5. Valve-in-Valve Procedures

Valve-in-valve procedures constitute a distinct clinical setting in which residual gradient, effective internal diameter, coronary-obstruction or sinus-sequestration risk, and future reintervention become particularly important. The included evidence was relatively limited and included comparative experience with ALLEGRA in failed surgical bioprostheses and smaller valve-in-valve subsets within self-expanding registries [60,66]. These findings should not be extrapolated directly to first-time native-valve TAVI.

### 3.6. Reporting Bias and Certainty of Evidence

Formal reporting-bias analyses and a formal GRADE certainty assessment were not performed. The structured qualitative endpoint-level appraisal described in the Methods is summarized in Table 3.

### 3.7. Representation of Valve Families and Device Generations

The included literature was concentrated in a relatively small number of recurrent valve families. The Edwards SAPIEN family and the Medtronic CoreValve/Evolut family were the most frequently represented platforms, each appearing in 25 included studies, and therefore dominated the balloon-expandable and self-expanding evidence streams, respectively. The ACURATE neo family showed moderate contemporary representation, whereas Portico and Navitor appeared less frequently. Myval and Hydra were represented in only a small number of studies, while ALLEGRA and the Vienna self-expandable systems appeared only once.

**Figure 2:**
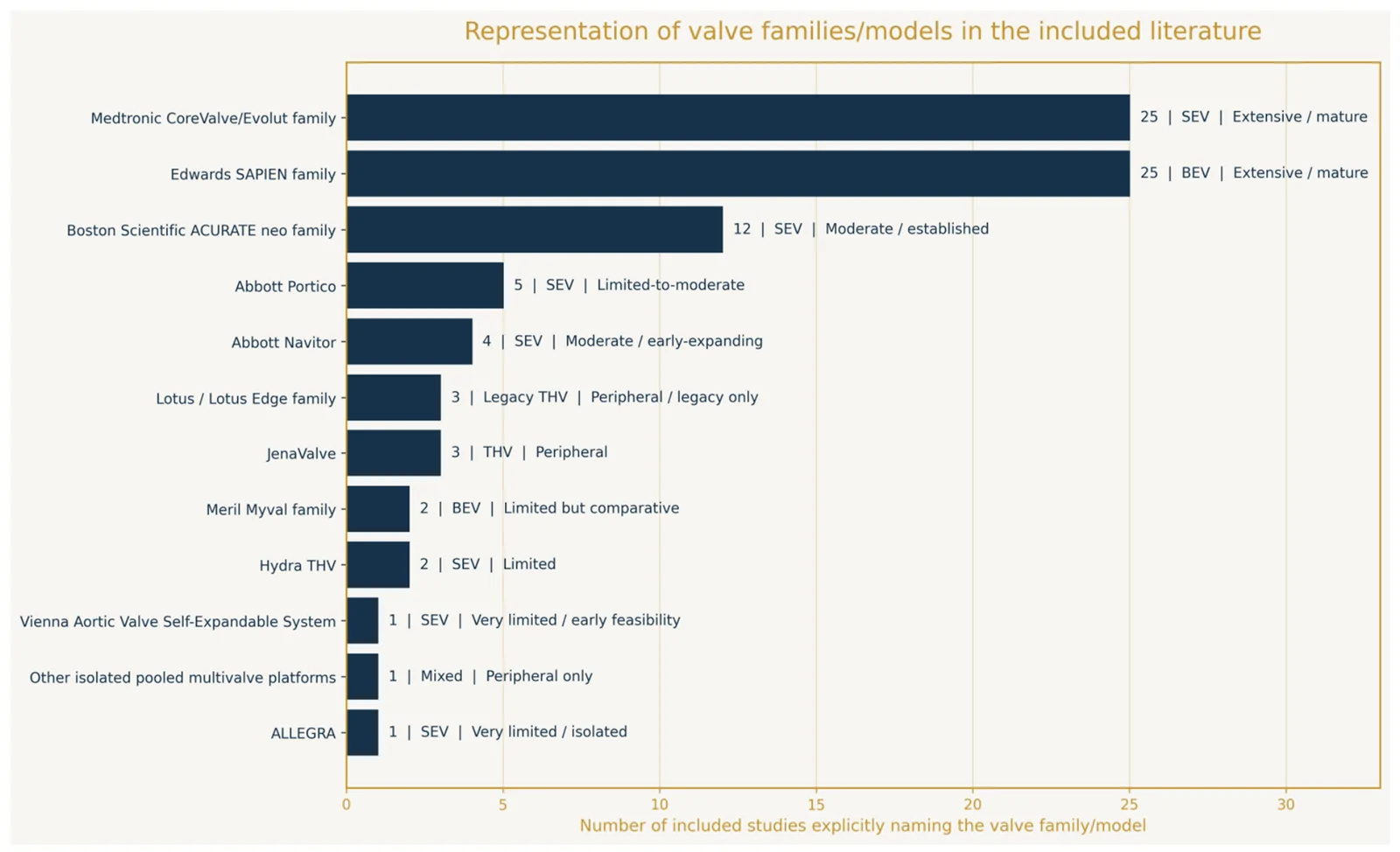
Representation of valve families/models in the included literature. Abbreviations: BEV, balloon-expandable valve; SEV, self-expandable valve; THV, transcatheter heart valve.

Generational breadth also differed across platforms. Prosthesis families such as the SAPIEN and CoreValve/Evolut lineages spanned the widest range of named device generations, whereas other families were represented by only one or two specific models. Legacy or isolated platforms, such as Lotus/Lotus Edge and other pooled multivalve devices, appeared mainly within descriptive or comparative datasets rather than as dedicated contemporary evidence streams. Overall, the mapped literature was anchored primarily in SAPIEN- and Evolut-based studies, with narrower evidence bases for the remaining valve families (Figure 2).

### 3.8. Coverage of the Prespecified Clinical and Anatomical Domains

Coverage across the prespecified clinical and anatomical domains was uneven (Table S7). The most extensively represented domains were permanent pacemaker implantation/conduction outcomes, paravalvular leak, and transvalvular hemodynamic performance, which were repeatedly reported across randomized trials, propensity-matched registries, and observational cohorts. Moderate coverage was available for bicuspid aortic valve anatomy, small annulus, and commissural alignment. Coronary access and coronary obstruction were addressed less often and were studied mainly through imaging- or reaccess-oriented series rather than datasets centered on adjudicated clinical obstruction events.

By contrast, several prespecified domains were supported by only limited evidence. Thrombocytopenia, acute kidney injury, endocarditis, horizontal aorta/aortic angulation, left ventricular systolic dysfunction, cusp calcification, balloon valvuloplasty strategy, and sex-related differences were each addressed in only a few studies and were often represented by single-center retrospective cohorts, exploratory subgroup analyses, or CT-based predictor studies. Across most domains, reporting concentrated on discharge, 30-day, and 1-year outcomes, with more limited long-term comparative data.

### 3.9. Cross-Cutting Evidence Gaps and Reporting Limitations

Several cross-cutting limitations affected interpretation of the mapped evidence base (Table S8). First, the literature was heavily weighted toward early procedural and early postprocedural outcomes, whereas long-term durability, late clinical consequences of residual valve-related findings, and sustained pacemaker dependency were less consistently reported. Second, definitions, grading thresholds, and ascertainment methods varied across studies, particularly for paravalvular leak, conduction outcomes, and anatomy-based imaging endpoints, which constrained direct cross-study comparison.

Third, much of the evidence outside the most frequently studied domains came from observational, single-center, retrospective, or post hoc analyses, thereby limiting causal inference and increasing vulnerability to confounding and selection bias. Fourth, several contemporary or emerging valve platforms were represented mainly by single-arm or early-feasibility datasets rather than robust head-to-head comparisons. Finally, phenotype-specific stratification remained limited across many studies, especially with respect to bicuspid phenotype, calcification pattern, coronary anatomy, sex-based analyses, and low-left-ventricular-ejection-fraction populations. Collectively, these limitations indicate that the current literature provides relatively strong information on recurrent early endpoints but leaves important uncertainty regarding underrepresented domains, standardized endpoint reporting, and long-term comparative performance.

## 4. Discussion

This systematic review synthesized 50 contemporary studies and indicates that the most clinically relevant differences between balloon-expandable and self-expanding transcatheter valve platforms reside less in crude survival alone and more in the balance among conduction outcomes, residual paravalvular leak, and hemodynamic performance [39,49,50]. An important implication of the available literature is that apparent clinical similarity at 30 days or 1 year should not be interpreted as true procedural equivalence. It must be noted that many platforms appear to succeed in terms of acceptable early safety through different trade-offs. A family may accept a higher pacemaker burden in order to present benefits in terms of lower gradients, whereas another may sacrifice some hemodynamic advantage to succeed achieving tighter sealing or more controlled deployment.

An important distinction in interpreting the present evidence is that findings observed with a particular valve model or generation should not automatically be extrapolated to the entire balloon-expandable or self-expanding class. Class-level interpretation is most defensible when a similar directional signal is reproduced across multiple independent valve families, generations, and comparative study designs. By contrast, findings confined to an individual platform iteration should remain generation-specific. This distinction is particularly relevant because iterative modifications in sealing skirts, frame geometry, delivery systems, implantation technique, and commissural-orientation strategies have altered the performance of contemporary devices. For example, improvements reported with SAPIEN 3 Ultra RESILIA relative to earlier SAPIEN iterations, Evolut FX and contemporary implantation workflows relative to earlier Evolut generations, and ACURATE neo2 relative to ACURATE neo represent generation-specific evolution rather than intrinsic properties of all BEV or SEV devices.

Consequently, studies combining earlier and contemporary generations were interpreted cautiously, and older-platform findings were not used alone to define the expected performance of currently used devices. Likewise, evidence from discontinued or legacy platforms was retained where required by the prespecified eligibility criteria for completeness of the systematic review, but was interpreted primarily as historical or mechanistic context rather than as a basis for contemporary prosthesis-selection recommendations. This generation-specific approach is particularly important in a rapidly evolving field in which improvements within a valve family may modify previously recognized trade-offs without necessarily changing the broader expansion mechanism.

Across the included literature, evidence was concentrated on permanent pacemaker implantation, paravalvular leak, and transvalvular gradients, whereas acute kidney injury, thrombocytopenia, sex-specific heterogeneity, endocarditis, and several anatomy-focused parameters were comparatively underexplored and were usually represented by single-center cohorts, incidental adverse-event reporting, or post hoc subgroup analyses [57,60,67,68]. This imbalance in the evidence base matters clinically. Pacemaker implantation, PVL, and gradients are attractive outcomes because they are frequent, readily measurable, and often available in registries or core-laboratory series. By contrast, platelet kinetics, renal effects, infective endocarditis, sex-specific interactions, and the behavior of devices in unusual anatomy such as bicuspid valves, horizontal aortas, very large annuli, or severe calcific asymmetry were less commonly addressed through prespecified comparative designs. In practice, this means that some of the most important “lifetime management” questions are informed not by broad randomized evidence but by smaller, more selective datasets [57,60,67,68].

Overall, supra-annular self-expanding valves were more often associated with lower residual gradient and less severe prosthesis-patient mismatch, while balloon-expandable valves more often showed lower residual regurgitation or lower pacemaker burden in selected comparative settings [49,50,51,52]. It must be noted that even this summary requires to be examined “under the microscope”. Lower gradients did not uniformly translate into superior survival, and more favorable sealing did not guarantee dominance across all anatomical contexts. Several studies suggested that the clinical relevance of postprocedural gradients depends on the specific population under study, particularly annular size and baseline ventricular function, whereas the clinical weight of PVL may be greater where calcification or expansion asymmetry is prominent. Similarly, the meaning of a higher pacemaker rate is not uniform. In fact, in some studies, it did not translate into excess late mortality, while on the other hand there were publications insisting that it remained an important marker of a less favorable procedural phenotype. The net effect is that platform choice appears best understood not as a search for a universally superior device class, but as a matching exercise between device mechanics and patient anatomy, baseline conduction vulnerability, and downstream coronary or redo-valve considerations [49,50,51,52].

### 4.1. Clinical Interpretation

**Figure 3:**
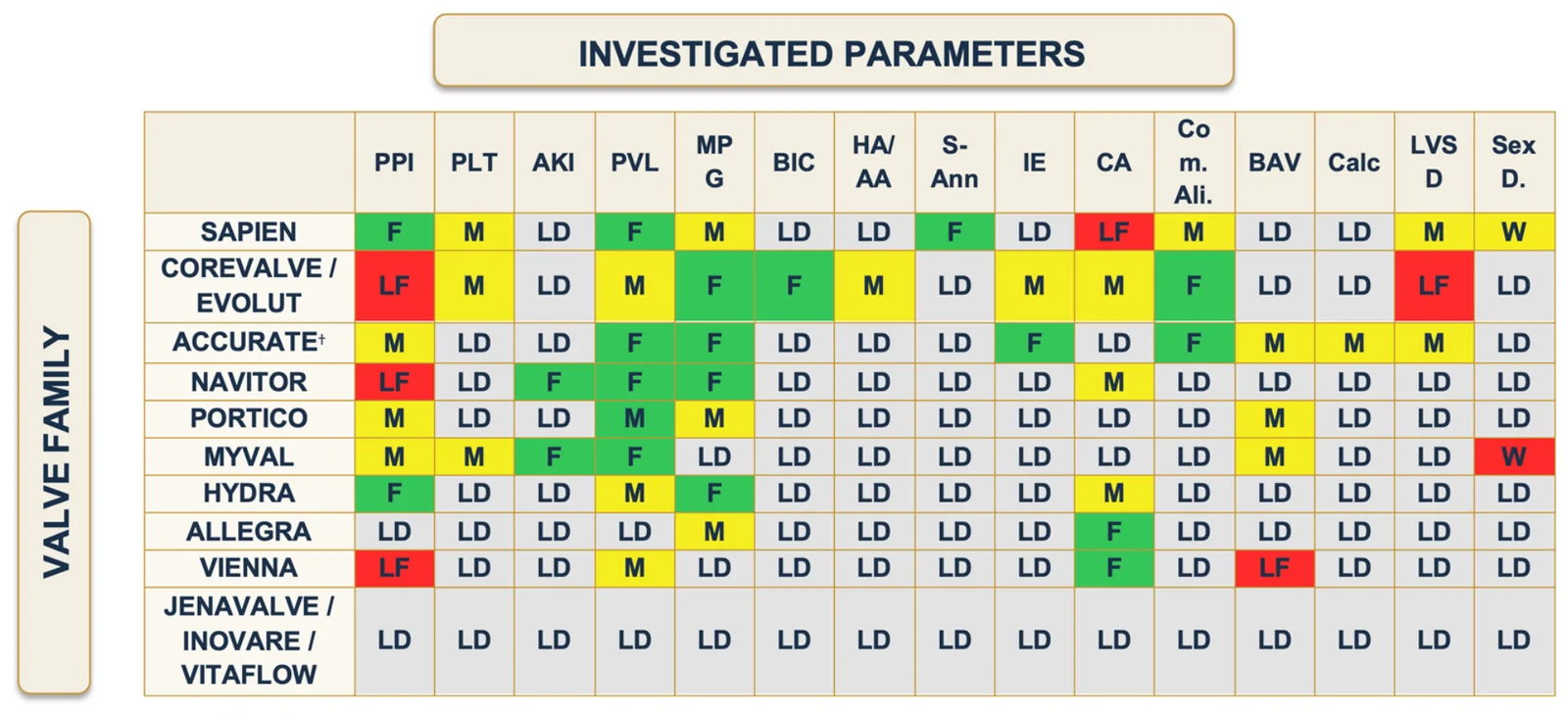
Valve family performance matrix across investigated parameters (Green = Favorable outcomes, Yellow = Mixed outcomes, Red = Less favorable outcomes, Grey = limited data). Abbreviations: PPI, Permanent Pacemaker Implantation; PLT, Thrombocytopenia/Platelet Drop; AKI, Acute Kidney Injury; PVL, Paravalvular Leak; MPG, Mean Pressure Gradient/Hemodynamics; BIC, Bicuspid Anatomy; HA/AA, Horizontal Aorta/Aortic Angulation; S-Ann, Small Annulus; IE, Infective Endocarditis; CA, Coronary Access/Obstruction; Com. Ali., Commissural Alignment; Calc, Native Cusp Calcification; LVSD, Left Ventricular Systolic Dysfunction; Sex D., Sex Difference; BAV, Balloon Valvuloplasty Before TAVI; TAVI, transcatheter aortic valve implantation; F, Favorable Outcomes; M, Mixed Outcomes; LF, Less Favorable Outcomes; LD, Limited Data; W, Less Favorable Outcomes for Women. † ACURATE neo/neo2 is retained for historical evidence mapping but is not included in contemporary device-selection recommendations following commercial discontinuation of the platform in 2025 [71].

The platform-specific clinical and valve-performance findings discussed below are summarized visually in Figure 3.

Among balloon-expandable, the Edwards SAPIEN family has the most extensive and mature evidence base in the present review. Across contemporary studies, its principal strengths were controlled deployment and a favorable sealing profile, while within-family evolution appears to have further modified performance. SAPIEN 3 Ultra RESILIA was associated with lower gradients and less residual PVL than earlier SAPIEN iterations, without an apparent increase in permanent pacemaker implantation, illustrating that clinically relevant differences may arise within a valve family rather than from expansion mechanism alone [69]. At the same time, the SAPIEN evidence does not support a uniformly favorable profile across all domains. Coronary and commissural considerations remain relevant to lifetime management, and dedicated evidence within the present dataset remains limited for bicuspid anatomy, extremely horizontal aorta, infective endocarditis, and several other less frequently investigated phenotypes [29,30,69].

The SAPIEN literature also illustrates why isolated surrogate outcomes should not be interpreted as clinical superiority. In patients with small annuli, higher early gradients or prosthesis–patient mismatch were not associated with worse 5-year outcomes within the SAPIEN 3 cohort, suggesting that the clinical importance of postprocedural hemodynamics depends on patient and anatomical context [57]. Similarly, permanent pacemaker implantation after SAPIEN 3 was not associated with increased 5-year mortality or repeat hospitalization in the relevant analysis, although persistent differences in left ventricular function suggest that conduction injury should not be regarded as clinically inconsequential [54]. Implantation depth, oversizing, prosthesis size, and calcification further emphasize that observed valve-family performance reflects an interaction between device design, anatomy, and procedural technique rather than valve class alone [54].

The Medtronic CoreValve/Evolut family showed its clearest strengths in supra-annular hemodynamics and in selected anatomically challenging settings, while conduction disturbance remained its principal recurring trade-off. Evidence in low-risk bicuspid anatomy demonstrated favorable clinical and hemodynamic outcomes with Evolut R/PRO, but these findings should not be extrapolated uncritically to all bicuspid phenotypes because procedural strategy and patient selection may contribute materially to the observed results [61]. More recent Evolut FX studies further demonstrate the importance of generation and technique: deliberate orientation and cusp-overlap implantation were associated with improved commissural alignment, implant-depth control, and contemporary pacing outcomes relative to earlier experience [31,32].

These refinements do not eliminate all of the limitations associated with a tall-frame supra-annular platform. RE-ACCESS 2 demonstrated that coronary cannulation may remain challenging despite systematic commissural-alignment strategies [58], while longer-term Evolut data confirm that conduction burden remains clinically relevant [59]. In patients with reduced left ventricular systolic function, lower gradients with self-expanding valves were accompanied by greater residual regurgitation and an observational signal toward higher adjusted mortality, which should be regarded as hypothesis-generating rather than causal [51]. Taken together, Evolut illustrates the central trade-off between favorable forward-flow hemodynamics and other considerations—including conduction vulnerability and future coronary interaction—and also demonstrates how implantation technique has become an integral component of contemporary device performance.

For the Boston Scientific ACURATE neo family, the available literature suggests that the ACURATE neo/neo2 experience provides one of the clearest examples of generation-specific evolution within the present evidence base. The principal limitation identified with the earlier ACURATE neo platform was residual PVL, whereas the expanded sealing skirt of neo2 was associated with a substantial reduction in moderate-or-greater regurgitation without an apparent deterioration in gradients or pacemaker outcomes [33,34]. The family also generated clinically relevant evidence concerning commissural alignment, coronary access, and implantation strategy [30,58,70]. Nevertheless, most neo2 evidence was observational or single-arm, important anatomical groups such as bicuspid valves were underrepresented, and favorable signals should therefore not be interpreted as evidence of superiority over contemporary alternative platforms.

ACURATE neo/neo2 studies were retained because they met the prespecified eligibility criteria and remain informative regarding the evolution of self-expanding valve design. However, following the 2025 decision to discontinue worldwide commercial sales of the ACURATE neo2 and ACURATE Prime systems [71], these findings are interpreted primarily as historical and mechanistic context and are not used to formulate recommendations concerning currently available prosthesis selection.

The Abbott Navitor platform showed encouraging contemporary performance with respect to sealing, gradients, and procedural success across the PORTICO NG and VANTAGE studies [36,37,72]. Its outer cuff and large-cell frame address two clinically important objectives—PVL reduction and preservation of future coronary access—while maintaining favorable hemodynamics. However, conduction disturbance remains an important limitation, and the available evidence is predominantly single-arm, with selective exclusion of challenging phenotypes such as bicuspid anatomy and severe LVOT calcification in key datasets. Navitor should therefore be regarded as a promising contemporary self-expanding platform with a favorable early performance profile, but not as evidence that the traditional hemodynamic–sealing–conduction trade-offs of self-expanding valves have been eliminated.

The Abbott Portico family is distinguished in the present evidence base primarily by procedural and delivery-system refinement. FlexNav reduced the delivery profile and was associated with favorable vascular and procedural outcomes, while the matched comparison with Myval showed broadly similar mortality, pacemaker implantation, and device-failure outcomes but greater reliance on adjunctive ballooning with Portico [35,38]. Small-annulus data also suggest potentially favorable hemodynamic performance, although these observations were not derived from a dedicated Portico randomized comparison [56]. Thus, the available evidence supports Portico as a procedurally refined and repositionable platform but does not establish a distinct class-level clinical advantage; its performance appears closely linked to implantation strategy and procedural optimization.

The Meril Myval family is notable because it is supported by randomized direct comparative evidence rather than only single-arm experience. In LANDMARK, Myval was non-inferior to both SAPIEN and Evolut for the early Valve Academic Research Consortium 3 (VARC-3) composite endpoint, with broadly comparable pacemaker and acute kidney injury outcomes and a hemodynamic profile that differed from both comparator families [39]. The matched comparison with Portico likewise demonstrated similar clinical outcomes with less frequent adjunctive ballooning [38]. These findings place Myval within contemporary head-to-head valve evaluation rather than solely as an emerging regional platform. Nevertheless, longer-term evidence and data in bicuspid anatomy, coronary reaccess, horizontal aorta, endocarditis, thrombocytopenia, and other important phenotype-specific domains remain limited, preventing equivalence with the mature evidence bases available for SAPIEN and Evolut [38,39].

For the Hydra platform, the evidence remains preliminary but directionally encouraging. The available CE-mark study demonstrated technically feasible implantation, favorable early gradients, and acceptable short-term valve performance, but it was a premarket single-arm investigation and therefore cannot establish comparative advantage [40]. Proposed benefits relating to recapturability, tortuous anatomy, prosthesis–patient mismatch, and coronary interaction remain mechanistically plausible but insufficiently validated, while evidence across several prespecified clinical and anatomical domains is absent or sparse. Hydra should therefore be interpreted as a developmental platform with encouraging early performance rather than as an established alternative supported by comparative outcome data.

Evidence for the remaining platforms was substantially more limited and should be interpreted according to the clinical context in which each device was studied. ALLEGRA was represented predominantly by valve-in-valve treatment of failed surgical bioprostheses, where favorable procedural and hemodynamic findings cannot be extrapolated directly to routine native-valve TAVI [66]. The Vienna self-expanding system was evaluated only in a small first-in-human feasibility study and therefore remains an early developmental platform despite favorable technical and hemodynamic observations [41]. JenaValve and Inovare lacked dedicated family-specific comparative clinical series within the included dataset, while VitaFlow/VitaFlow Liberty was not represented by an eligible dedicated comparator study. These platforms were therefore retained in the evidence map for completeness but were not used to support broad BEV-versus-SEV conclusions or contemporary platform superiority.

### 4.2. Strengths and Limitations

The findings of this discussion should therefore be interpreted in light of the underlying evidence structure. A strength of the present review is its focus on a contemporary time window and on named valve families or model generations, which makes the synthesis more relevant to current practice than older platform-overview reviews. This family-level approach is especially valuable in a field where iterative redesign has changed the practical meaning of platform choice. A review that combines SAPIEN XT, early SAPIEN 3, SAPIEN 3 Ultra, and SAPIEN 3 Ultra RESILIA into a single BEV construct, or that treats CoreValve, Evolut R, Evolut PRO, and Evolut FX as interchangeable, risks obscuring clinically important gains in sealing, delivery, commissural management, and conduction performance.

However, most of the included studies were observational, many were retrospective, follow-up was frequently limited to discharge, 30 days, or 1 year, and outcome ascertainment was not standardized across reports. These limitations increase the risk of residual confounding, selection bias, and misleading comparisons between studies that applied different clinical thresholds, imaging protocols, and echocardiographic adjudication methods [23]. Even within apparently comparable outcome domains, definitions and ascertainment were inconsistent. Pacemaker implantation, for example, may vary according to local decision thresholds and conduction-monitoring practices; PVL may be assessed by angiography, echocardiography, or quantitative videodensitometry; and even device success is not fully comparable across studies using different protocol eras or VARC iterations. In addition, many device-family datasets selectively excluded patients with bicuspid valves, prior bioprostheses, severe LVOT calcification, severe left ventricular dysfunction, or hostile vascular anatomy. Consequently, favorable findings in some series may partly reflect patient selection rather than platform performance alone. The key implication is that comparisons across studies should remain cautious, even when the numerical differences appear substantial.

The limitations of the review process should also be stated explicitly. The search was restricted to the database “PubMed”, the review window covered publications from early 2020 to late 2025, and the working dataset was further limited to freely accessible full-text articles. Together, these criteria may have introduced retrieval bias and reduced the capture of otherwise eligible contemporary studies. This is particularly important in a rapidly evolving prosthesis field, in which clinically relevant data may first appear preliminary in conference presentations, subscription-based journals, or industry-supported reports before later maturing into complete publications. The free-full-text restriction may also have preferentially captured open-access registry studies and investigator-led reports while excluding relevant comparator analyses. The review was not prospectively registered because it was initially conducted under a written M.D. thesis protocol developed before commencement of the literature search rather than through a prospective public review registry. The prespecified protocol is available from the corresponding author upon reasonable request, and subsequent methodological deviations or refinements are reported transparently in Supplementary Table S3; nevertheless, the absence of prospective public registration remains a limitation. In addition, because the evidence map was dominated by studies focused on named platforms rather than standardized head-to-head datasets, the absence of evidence for a given device family in a specific phenotype should not be interpreted as evidence of inferiority. In many cases, it more likely reflects underrepresentation.

The decision not to perform a meta-analysis was methodologically appropriate given the heterogeneity in study designs, devices, outcome definitions, and reporting intervals. Nonetheless, it also means that the conclusions remain interpretive rather than quantitatively pooled. For that reason, this review is best understood as a structured contemporary evidence map rather than a definitive estimate of pooled comparative effect. This framing is less a weakness than a disciplined acknowledgment of what the current literature can and cannot support. A pooled estimate combining different device generations, mixed-risk populations, varying anatomical exclusions, and inconsistent endpoint frameworks might have created an impression of precision without meaningfully improving validity. The present approach instead prioritizes clinical interpretability and device-specific context over a potentially misleading numerical synthesis. These considerations also underscore the importance of transparent reporting when synthesis is performed without meta-analysis, as emphasized by the Synthesis without Meta-analysis (SWiM) reporting guideline reporting guideline [73].

### 4.3. Implications for Practice and Future Research

From a practical perspective, the present evidence supports a phenotype- and anatomy-informed approach in which prosthesis selection begins with the dominant patient-specific constraint rather than with a universal preference for balloon- or self-expanding technology. In patients with a small annulus or a high anticipated risk of prosthesis–patient mismatch, supra-annular hemodynamics may be advantageous, although the clinical importance of lower residual gradients should be interpreted in the context of age, ventricular function, and expected longevity. Conversely, in patients with baseline conduction vulnerability, including pre-existing right bundle branch block or anatomical features associated with conduction-system interaction, both valve design and implantation depth or technique should be considered; in patients with an existing permanent pacemaker, the clinical relevance of new pacing requirements may be less discriminatory. Heavy annular or LVOT calcification, asymmetric calcific burden, bicuspid morphology, very large annuli, and marked aortic angulation or horizontal aorta require individualized CT-based assessment because available evidence is more platform- and anatomy-specific than class-wide. In younger patients or those with established coronary artery disease or a high likelihood of future coronary intervention, frame height, stent-cell architecture, commissural orientation, coronary reaccess, and the feasibility of future TAVI-in-TAVI should assume greater importance. Similarly, valve-in-valve procedures require consideration of the surgical bioprosthesis internal diameter, expected residual gradient, and the risk of coronary obstruction or sinus sequestration. These factors should not be interpreted as rigid indications for a particular valve class; rather, they illustrate how the balance between hemodynamics, sealing, conduction risk, coronary accessibility, and lifetime reintervention strategy should guide individualized Heart Team selection.

Beyond anatomical and device-specific considerations, dynamic functional assessment may provide complementary information in selected patients before and after TAVI. Exercise stress echocardiography (ESE), although outside the prespecified device-comparison scope of this review, may help characterize the functional consequences of aortic stenosis beyond resting measurements and contribute additional prognostic information during patient assessment [74]. Following TAVI, exercise-based echocardiographic evaluation may also provide complementary information regarding functional recovery and subsequent clinical risk beyond resting clinical and echocardiographic parameters [75]. These emerging data do not currently support preferential selection of a particular BEV or SEV platform; rather, they suggest that dynamic functional assessment may complement anatomy-based evaluation and individualized follow-up when clinically appropriate.

**Figure 4:**
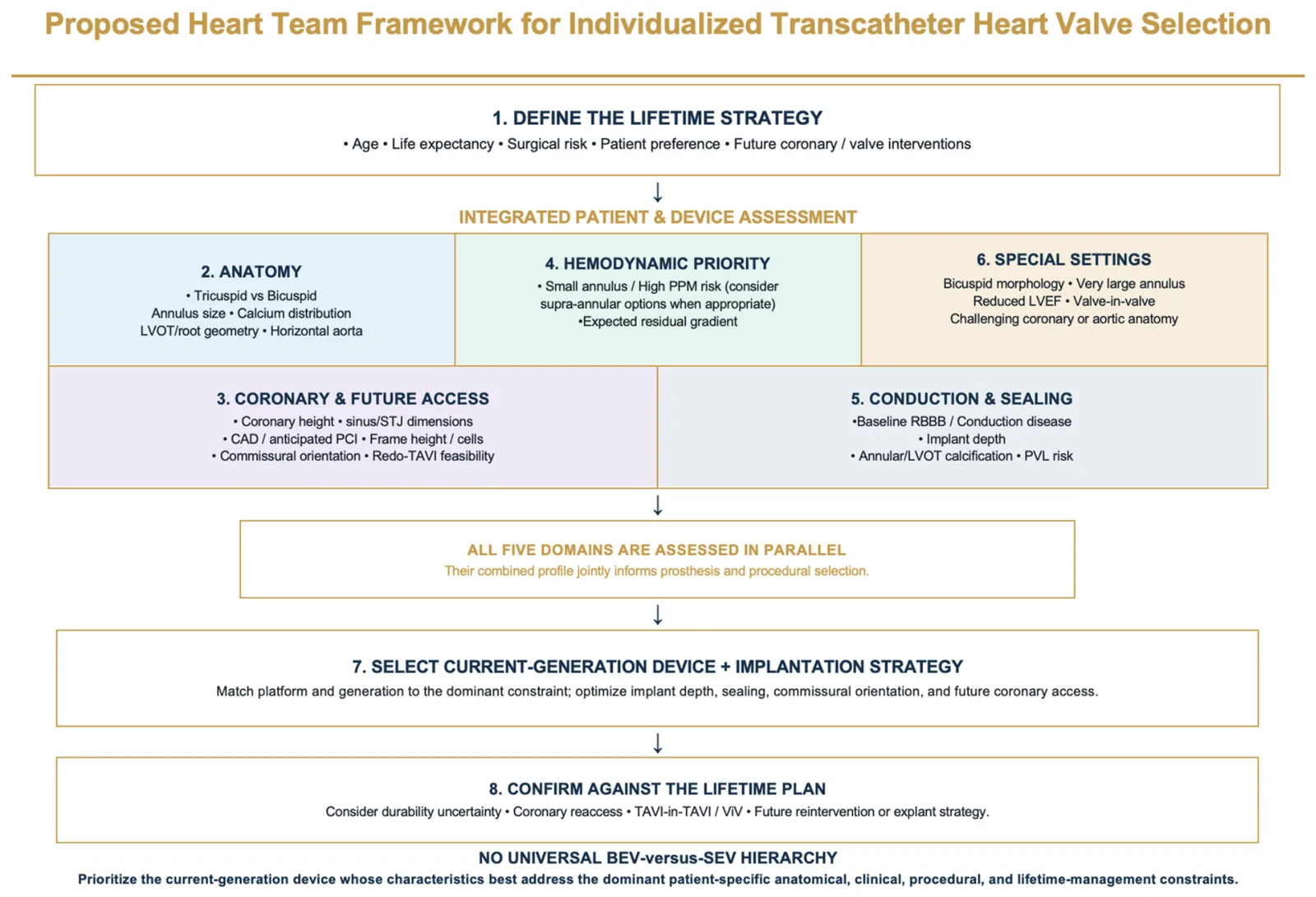
Proposed Heart Team framework for individualized transcatheter heart valve selection. Abbreviations: BEV, balloon-expandable valve; SEV, self-expandable valve; CAD, coronary artery disease; PCI, percutaneous coronary intervention; PPM, prosthesis-patient mismatch; PVL, paravalvular leak; RBBB, right bundle branch block; LVOT, left ventricular outflow tract; LVEF, left ventricular ejection fraction; STJ, sinotubular junction; TAVI, transcatheter aortic valve implantation; ViV, valve-in-valve. Framework derived from qualitative evidence synthesis and intended to support, not replace, individualized Heart Team judgment.

Lifetime management is increasingly relevant as TAVI expands to younger patients with longer anticipated survival. The available evidence in this review does not establish a durable BEV-versus-SEV advantage with respect to structural valve degeneration or late reintervention, because long-term comparative follow-up remains limited and uneven across valve families and generations. Initial prosthesis selection should therefore anticipate not only index procedural performance but also the feasibility of future coronary access, TAVI-in-TAVI, the potential hemodynamic consequences of a second transcatheter valve, and, where relevant, the complexity of eventual surgical explantation. Frame height, commissural orientation, implantation depth, residual sinus dimensions, and the anticipated neo-skirt after redo-TAVI may consequently become increasingly important in patients with long life expectancy. At present, these lifetime-management considerations support individualized prospective planning rather than preferential selection of BEV or SEV on the basis of demonstrated durability superiority. A practical Heart Team framework integrating these anatomical, clinical, procedural, and lifetime-management considerations is summarized in Figure 4.

Future research should also move beyond the now-familiar pacemaker/PVL/gradient triad. Longer-term comparative studies should prioritize structural valve deterioration, reintervention, coronary reaccess, valve thrombosis, endocarditis, sex-based anatomical interactions, ventricular recovery in low-flow or low-EF states, platelet behavior, renal safety, and performance in anatomically complex phenotypes such as bicuspid valves, severe leaflet calcification, horizontal aortas, or degenerated bioprostheses. Equally important is the need to preserve generational specificity. Trials and registries that compare a new platform to a heterogeneous “contemporary valve” control arm remain useful, but they should not obscure model-level differences that may be decisive in practice. Standardized core-laboratory imaging, harmonized endpoint definitions, and longer surveillance for reintervention and coronary access outcomes will be particularly important as TAVI continues its shift toward younger and lower-risk populations.

## 5. Conclusions

This systematic review suggests that the comparison between balloon-expandable and self-expanding transcatheter aortic valve platforms cannot be reduced to a simple binary ranking. Across the included contemporary studies, the most consistent differences were observed in permanent pacemaker implantation, residual paravalvular leak, and postprocedural hemodynamic performance rather than in a reproducible survival advantage for either platform. Within the limitations of the mapped literature, supra-annular self-expanding valves appeared more often to be associated with lower residual gradients and a lower burden of prosthesis-patient mismatch, whereas balloon-expandable valves appeared more often to be associated with more favorable sealing profiles or lower pacemaker burden in selected comparative settings.

Taken together, the available contemporary evidence indicates that valve selection in TAVI is better understood as an individualized, phenotype-informed decision rather than a uniform platform preference. The present findings should therefore be interpreted as a structured synthesis of current patterns in the literature, not as definitive proof of platform superiority. In clinical practice, these patterns may help inform Heart Team decision-making by considering device design alongside annular and root anatomy, baseline conduction vulnerability, implantation strategy, and lifetime management priorities. However, because the evidence base was heterogeneous, predominantly observational, selectively retrieved, and not quantitatively pooled, these inferences remain provisional and should be refined by further head-to-head, methodologically robust, and longer-term comparative studies.

A final practical conclusion emerging from the present dataset is that the clinically relevant unit of decision-making is increasingly the named valve family and, often, the specific generation within that family rather than the broad expansion mechanism alone. Contemporary TAVI practice should therefore integrate platform- and generation-specific characteristics with anatomy, conduction risk, coronary considerations, hemodynamic priorities, and anticipated lifetime management. In this context, the available evidence does not identify a single universal “Queen of Hearts” among contemporary TAVI platforms; rather, the optimal prosthesis depends on matching current-generation device characteristics to the individual patient.

## Figures and Tables

**Table 1:** Stepwise PubMed search strategy and representative keywords used for study identification ^1,2^.

Search Step	Search Block/Logic	Representative Keywords	Records
#1	Balloon-expandable platform terms	balloon-expandable, “balloon expandable”, SAPIEN, Edwards, Myval, Meril, INOVARE	12,016
#2	Self-expandable platform terms	self-expandable, “self-expandable”, Evolut, CoreValve, Navitor, Portico, ACURATE, VitaFlow, Hydra, Vienna	18,571
#3	Procedure terms	“Transcatheter Aortic Valve Replacement” [MeSH], transcatheter aortic valve replacement, transcatheter aortic valve implantation, TAVR, TAVI	23,409
#4	Combined device and procedure block	(#1 OR #2) AND #3	4616
#5	Prespecified clinical and anatomical factor block	Pacemaking, conduction disturbance, LBBB, RBBB, thrombocytopenia, platelet number, acute kidney injury, AKI, paravalvular leak, mean pressure gradient, bicuspid valve, horizontal aorta, aortic angulation, small annulus, endocarditis, coronary artery obstruction, commissural alignment, balloon valvuloplasty, cusp calcification, left ventricular systolic dysfunction, sex difference	1188
#6	Exclusion for non-target access routes	transcarotid access, transapical access, substernal access, transcaval access, transaortic access	1175
#7	Restriction to human studies	humans [MeSH Terms]	892
#8	Exclusion of review-level publications	review, systematic review, meta-analysis, network meta-analysis	802
#9	Restriction to centre-based original reports	“Multicenter study” [pt], multicenter, multi-center, single-center, single center	270
#10	Limit by publication date and free full-text	“2020/01/01” to “2025/12/31” [Date-Publication]	64

^1^ Note. Representative terms are shown for readability. The exact final PubMed search history (10) is reproduced below in full. ^2^ Abbreviations: AKI, Acute Kidney Injury; LBBB, Left Bundle Branch Block; MeSH, Medical Subject Headings; pt, publication type; RBBB, Right Bundle Branch Block; TAVI, Transcatheter Aortic Valve Implantation; TAVR, Transcatheter Aortic Valve Replacement.

**Table 2:** Study selection summary.

Screening Step	Number of Records	Notes
Records identified through database search	64	PubMed only; 2020–2025; exported 25 January 2026—Full-text access freely available
Records screened	64	Title/abstract screening
Records excluded during screening	0	No title/abstract exclusions
Reports sought for retrieval	64	Full texts sought for all screened records
Reports not retrieved	1	Full-text access required payment
Reports assessed for eligibility	63	Full-text review
Reports excluded after eligibility review	13	Outside year range (1); non-target access (4); mechanical/mixed platforms (4); ineligible design/protocol (2); not aligned with aim (2)
Studies included in qualitative synthesis	50	Final included study set

**Table 3:** Qualitative strength and consistency of evidence across principal outcome domains.

Outcome Domain	Predominant Evidence	Overall Comparative Signal	Consistency	Relative Evidence Strength
Mortality	Randomized follow-up and adjusted/propensity-matched comparative cohorts	No reproducible class-level survival advantage for BEV or SEV	Mixed across populations	Moderate
Stroke	Randomized and observational comparative studies, generally secondary endpoints	No universal platform advantage; individual trial-specific differences reported	Mixed	Moderate
PPI/conduction disturbances	Randomized, adjusted comparative, registry, and dedicated conduction studies	Higher pacing burden with several SEV platforms, but strongly modified by generation and implantation technique	Relatively consistent with important modifiers	Stronger
PVL/regurgitation	Randomized, adjusted observational, registry, and imaging studies	BEV frequently associated with more favorable sealing; newer SEV generations have narrowed the difference	Relatively consistent but generation-dependent	Stronger
Gradients/EOA/PPM	Randomized and multiple comparative observational studies	Supra-annular SEV consistently associated with lower gradients and less PPM, particularly in small annuli	Relatively consistent	Stronger
Coronary access/commissural alignment	Predominantly CT-, angiography-, and procedure-based observational studies	Tall-frame supra-annular systems may create greater reaccess challenges; alignment improves but does not eliminate this issue	Moderately consistent mechanistically	Limited–Moderate
Valve thrombosis/durability	Selected longitudinal cohorts and trial-derived follow-up	Insufficient evidence for durable superiority of either platform	Limited	Limited
Reintervention	Selected long-term cohorts; relatively low event rates	No consistent platform difference demonstrated	Limited	Limited

Abbreviations: BEV, balloon-expandable valve; SEV, self-expandable valve; PPI, permanent pacemaker implantation; PVL, paravalvular leak; EOA, effective orifice area; PPM, prosthesis–patient mismatch; CT, computed tomography; GRADE, Grading of Recommendations Assessment, Development and Evaluation.Note: Relative evidence strength represents a qualitative within-review appraisal based on study design, risk of bias, consistency, directness, and breadth/duration of follow-up. It is not a formal GRADE certainty rating.

## Data Availability

No new data were created or analyzed in this study. Data sharing is not applicable to this article. All information discussed in this review is available from the cited published literature.

## Supplementary Materials

The following supporting information can be downloaded at: https://www.mdpi.com/article/10.3390/jcm15187224/s1, Table S1, exact final PubMed search query; Table S2, study-level characteristics of the included studies; Table S3, deviations from and methodological refinements to the original thesis protocol; Table S4, RoB 2 assessment of randomized-trial-derived studies; Table S5, MINORS assessment of non-randomized studies; Table S6, representation of valve families/models in the included literature; Table S7, mapping of the included literature across prespecified domains and factors; Table S8, identified evidence gaps and reporting limitations. The completed PRISMA 2020 checklist is submitted separately. References [76,77,78,79,80,81,82,83,84,85,86,87,88,89] are cited in the Supplementary Materials.

## Abbreviations

The following abbreviations are used in this manuscript:

BEV	Balloon-Expandable Valve
CE	European Conformity
CT	Computed Tomography
EF	Ejection Fraction
LVOT	Left Ventricular Outflow Tract
MINORS	Methodological Index for Non-Randomized Studies
PPM	Prosthesis–Patient Mismatch
PPI	Permanent Pacemaker Implantation
PRISMA	Preferred Reporting Items for Systematic Reviews and Meta-Analyses
PVL	Paravalvular Leak
RoB	Risk of Bias
SAVR	Surgical Aortic Valve Replacement
SEV	Self-Expandable Valve
TAVI	Transcatheter Aortic Valve Implantation
TAVR	Transcatheter Aortic Valve Replacement
VARC	Valve Academic Research Consortium
