# Supplementary material for: Balloon-Expandable Versus Self-Expandable Transcatheter Aortic Valve Implantation Devices: A Contemporary Systematic Review
Source: J Clin Med. 2026 Sep 17;15(18):7224. doi: 10.3390/jcm15187224 (PMC13607979; doi:10.3390/jcm15187224)
Supplement: Supplementary file 1 [file jcm-15-07224-s001.zip › PRISMA 2020 Checklist - JCM updated.pdf]

PRISMA 2020 Checklist

Section and Topic	Item #	Checklist item	Location where item is reported
**TITLE**			
Title	1	Identify the report as a systematic review.	Title page (p. 1): article type and title identify the report as a systematic review.
**ABSTRACT**			
Abstract	2	See the PRISMA 2020 for Abstracts checklist.	Structured Abstract (p. 2)
**INTRODUCTION**			
Rationale	3	Describe the rationale for the review in the context of existing knowledge.	Section 1, Introduction: "Theoretical Background", "Existing Knowledge", and "Description of the Problem".
Objectives	4	Provide an explicit statement of the objective(s) or question(s) the review addresses.	Section 1, "Purpose and Specific Objectives".
**METHODS**			
Eligibility criteria	5	Specify the inclusion and exclusion criteria for the review and how studies were grouped for the syntheses.	Section 2, "Eligibility criteria"; Section 2, "Data synthesis" (grouping by valve platform and prespecified clinical/anatomical domain).
Information sources	6	Specify all databases, registers, websites, organisations, reference lists and other sources searched or consulted to identify studies. Specify the date when each source was last searched or consulted.	Section 2, "Description of search strategy" (PubMed only; searched 25 January 2026); "Study selection process". Supplementary Table S3 reports that planned reference-list screening was not undertaken.
Search strategy	7	Present the full search strategies for all databases, registers and websites, including any filters and limits used.	Section 2, "Description of search strategy" and "Exact Final PubMed Query"; Tables 1 and Supplementary S1 (complete strategy, field tags, filters, and limits).
Selection process	8	Specify the methods used to decide whether a study met the inclusion criteria of the review, including how many reviewers screened each record and each report retrieved, whether they worked independently, and if applicable, details of automation tools used in the process.	Section 2, "Study selection process" (reviewer roles, verification, disagreement resolution, and no automation tools).
Data collection process	9	Specify the methods used to collect data from reports, including how many reviewers collected data from each report, whether they worked independently, any processes for obtaining or confirming data from study investigators, and if applicable, details of automation tools used in the process.	Section 2, "Data extraction" (extractor, verifier/curator, disagreement resolution, handling of missing information, and no investigator contact).
Data items	10a	List and define all outcomes for which data were sought. Specify whether all results that were compatible with each outcome domain in each study were sought (e.g. for all measures, time points, analyses), and if not, the methods used to decide which results to collect.	Section 2, "Data extraction" (outcome and clinical/procedural/anatomical domains; all relevant follow-up points; rule for multiple adjusted estimates).
	10b	List and define all other variables for which data were sought (e.g. participant and intervention characteristics, funding sources). Describe any assumptions made about any missing or unclear information.	Section 2, "Data extraction" (author/year, setting, design, centre type, sample size, valve family/model, comparator, population/subgroup, follow-up, and outcomes; unclear data recorded as not reported).
Study risk of bias assessment	11	Specify the methods used to assess risk of bias in the included studies, including details of the tool(s) used, how many reviewers assessed each study and whether they worked independently, and if applicable, details of automation tools used in the process.	Section 2, "Quality assessment"/risk-of-bias methods (RoB 2 and MINORS; reviewer verification and consensus procedure).
Effect measures	12	Specify for each outcome the effect measure(s) (e.g. risk ratio, mean difference) used in the synthesis or presentation of results.	Section 2, "Effect measures".
Synthesis methods	13a	Describe the processes used to decide which studies were eligible for each synthesis (e.g. tabulating the study intervention characteristics and comparing against the planned groups for each synthesis (item #5)).	Section 2, "Eligibility criteria" and "Data synthesis"; Supplementary Table S2 maps studies by valve platform,

PRISMA 2020 Checklist

Section and Topic	Item #	Checklist item	Location where item is reported
			comparison type, population/domain, and follow-up.
	13b	Describe any methods required to prepare the data for presentation or synthesis, such as handling of missing summary statistics, or data conversions.	Section 2, “Data synthesis” (missing summary statistics were not imputed and no data conversions were undertaken).
	13c	Describe any methods used to tabulate or visually display results of individual studies and syntheses.	Section 2, “Data synthesis”; Supplementary Table S2 presents detailed study-level data, while Table 3, Figures 2–4, and Supplementary Tables S6–S8 present synthesis-level and visual summaries of the evidence..
	13d	Describe any methods used to synthesize results and provide a rationale for the choice(s). If meta-analysis was performed, describe the model(s), method(s) to identify the presence and extent of statistical heterogeneity, and software package(s) used.	Section 2, “Data synthesis” (narrative synthesis and rationale for not performing meta-analysis).
	13e	Describe any methods used to explore possible causes of heterogeneity among study results (e.g. subgroup analysis, meta-regression).	Not applicable: no statistical subgroup analysis or meta-regression was performed. See Section 2, “Data synthesis”.
	13f	Describe any sensitivity analyses conducted to assess robustness of the synthesized results.	Not applicable: no sensitivity analyses were performed. See Section 2, “Data synthesis”.
Reporting bias assessment	14	Describe any methods used to assess risk of bias due to missing results in a synthesis (arising from reporting biases).	Section 2, “Assessment of Reporting Bias”.
Certainty assessment	15	Describe any methods used to assess certainty (or confidence) in the body of evidence for an outcome.	Section 2, “Certainty of Evidence”.
**RESULTS**			
Study selection	16a	Describe the results of the search and selection process, from the number of records identified in the search to the number of studies included in the review, ideally using a flow diagram.	Section 3, “Study Selection”; Table 2; Figure 1. One report was not retrieved because full-text access required payment.
	16b	Cite studies that might appear to meet the inclusion criteria, but which were excluded, and explain why they were excluded.	Section 3, “Study Selection”; Table 2. Thirteen reports were excluded following full-text assessment, with the reasons summarized by exclusion category. Individual excluded reports were not separately tabulated.
Study characteristics	17	Cite each included study and present its characteristics.	Section 3, “General characteristics of the included studies”; Table S2 (all included studies cited and characterised).
Risk of bias in studies	18	Present assessments of risk of bias for each included study.	Section 3, “Methodological quality and risk-of-bias assessment of the included studies”; Tables S4 and S5.
Results of individual studies	19	For all outcomes, present, for each study: (a) summary statistics for each group (where appropriate) and (b) an effect estimate and its precision (e.g. confidence/credible interval), ideally using structured tables or plots.	Supplementary Table S2 and the domain-specific narrative Results sections present study-level summary data and reported effect estimates/precision where available.
Results of syntheses	20a	For each synthesis, briefly summarise the characteristics and risk of bias among contributing studies.	Section 3, “General characteristics…”, “Methodological quality and risk-of-bias…”, and domain-specific syntheses; Section 3, including “Evidence Structure and Methodological Quality” and the domain-specific syntheses; Table 3; Supplementary Tables S2 and S4–S8; and Figures 2–4..

PRISMA 2020 Checklist

Section and Topic	Item #	Checklist item	Location where item is reported
	20b	Present results of all statistical syntheses conducted. If meta-analysis was done, present for each the summary estimate and its precision (e.g. confidence/credible interval) and measures of statistical heterogeneity. If comparing groups, describe the direction of the effect.	Not applicable: no statistical synthesis or meta-analysis was conducted. Narrative synthesis is reported in Section 3, Table 3, Figures 2–4, and Supplementary Tables S2 and S6–S8..
	20c	Present results of all investigations of possible causes of heterogeneity among study results.	Not applicable: no formal subgroup analysis or meta-regression was conducted. See Section 2, “Data synthesis”.
	20d	Present results of all sensitivity analyses conducted to assess the robustness of the synthesized results.	Not applicable: no sensitivity analyses were conducted. See Section 2, “Data synthesis”.
Reporting biases	21	Present assessments of risk of bias due to missing results (arising from reporting biases) for each synthesis assessed.	Not applicable: no formal reporting-bias assessment was performed. Rationale is reported in Section 2, “Assessment of Reporting Bias”, and Section 3, “Reporting Bias and Certainty of Evidence”.
Certainty of evidence	22	Present assessments of certainty (or confidence) in the body of evidence for each outcome assessed.	Section 2, “Certainty of Evidence”; Section 3, “Reporting Bias and Certainty of Evidence”; Table 3. A formal GRADE assessment was not performed; Table 3 presents the structured qualitative endpoint-level evidence-strength appraisal.
**DISCUSSION**			
Discussion	23a	Provide a general interpretation of the results in the context of other evidence.	Section 4, “Discussion”, including “Clinical interpretation”; Section 5, “Conclusions”.
	23b	Discuss any limitations of the evidence included in the review.	Section 4, “Strengths and limitations” (limitations of the included evidence).
	23c	Discuss any limitations of the review processes used.	Section 4, “Strengths and limitations” (limitations of the review methods and processes).
	23d	Discuss implications of the results for practice, policy, and future research.	Section 4, “Implications for practice and future research”; Section 5, “Conclusions”.
**OTHER INFORMATION**			
Registration and protocol	24a	Provide registration information for the review, including register name and registration number, or state that the review was not registered.	Section 2, “Registration and Protocol” (states that the review was not prospectively registered).
	24b	Indicate where the review protocol can be accessed, or state that a protocol was not prepared.	Section 2, “Registration and Protocol” (written thesis protocol available from the corresponding author upon reasonable request).
	24c	Describe and explain any amendments to information provided at registration or in the protocol.	Section 2, “Registration and Protocol”; Supplementary Table S3 (deviations and methodological refinements, with explanations).
Support	25	Describe sources of financial or non-financial support for the review, and the role of the funders or sponsors in the review.	Back matter, “Funding” (no external funding); “Acknowledgments” (non-financial methodological guidance).
Competing interests	26	Declare any competing interests of review authors.	Back matter, “Conflicts of Interest”.

PRISMA

PRISMA 2020 Checklist

Section and Topic	Item #	Checklist item	Location where item is reported
Availability of data, code and other materials	27	Report which of the following are publicly available and where they can be found: template data collection forms; data extracted from included studies; data used for all analyses; analytic code; any other materials used in the review.	Back matter, “Data Availability Statement”; extracted study-level information is presented in Supplementary Table S2, with additional evidence mapping provided in Supplementary Tables S6–S8. No separate dataset or analytic code was generated or made publicly available.

From: Page MJ, McKenzie JE, Bossuyt PM, Boutron I, Hoffmann TC, Mulrow CD, et al. The PRISMA 2020 statement: an updated guideline for reporting systematic reviews. BMJ 2021;372:n71. doi: 10.1136/bmj.n71.
This work is licensed under CC BY 4.0. To view a copy of this license, visit https://creativecommons.org/licenses/by/4.0/
