# Supplementary material for: Balloon-Expandable Versus Self-Expandable Transcatheter Aortic Valve Implantation Devices: A Contemporary Systematic Review
Source: J Clin Med. 2026 Sep 17;15(18):7224. doi: 10.3390/jcm15187224 (PMC13607979; doi:10.3390/jcm15187224)
Supplement: Supplementary file 1 [file jcm-15-07224-s001.zip › Supplementary_list.pdf]

Journal of Clinical Medicine

Systematic Review

Balloon-Expandable Versus Self-Expandable Transcatheter Aortic Valve Implantation Devices: A Contemporary Systematic Review

Ioannis Apergis [1,†], Georgios P. Georghiou [1,2,*,†], Panos Georghiou [3], Efthalia Emmanouela Chrisofos [1], Filippos Triposkiadis [1] and Konstantinos Lampropoulos [1]

1 School of Medicine, European University Cyprus, Nicosia 2404, Cyprus; japergis1@icloud.com (I.A.); thaliachrisofou@gmail.com (E.E.C.); ftriposkiadis@gmail.com (F.T.); lampropouloskon@gmail.com (K.L.)
2 Department of Surgery, Gray Faculty of Medical and Health Sciences, Tel Aviv University, Tel Aviv 6997801, Israel
3 Barts and The London School of Medicine and Dentistry, Queen Mary University of London, Turner St., London E1 2AD, UK; panos.georghiou@gmail.com
* Correspondence: geo.georgiou@euc.ac.cy
† These authors contributed equally to this work.

Supplementary materials

Table S1. Exact final PubMed search query

```
(
	(
		"balloon-expandable"[Title/Abstract] OR "balloon expandable"[Title/Abstract] OR
		"SAPIEN"[Title/Abstract] OR "Edwards"[Title/Abstract] OR "Myval"[Title/Abstract] OR
		"Meril"[Title/Abstract] OR "INOVARE"[Title/Abstract] OR
		"self-expandable"[Title/Abstract] OR "self expandable"[Title/Abstract] OR
		"Evolut"[Title/Abstract] OR "CoreValve"[Title/Abstract] OR "Navitor"[Title/Abstract] OR
		"Portico"[Title/Abstract] OR "ACURATE"[Title/Abstract] OR "VitaFlow"[Title/Abstract] OR
		"Hydra"[Title/Abstract] OR "Vienna"[Title/Abstract]
	)
	AND
	(
		"Transcatheter Aortic Valve Replacement"[MeSH Terms] OR
		"transcatheter aortic valve replacement"[Title/Abstract] OR
		"transcatheter aortic valve implantation"[Title/Abstract] OR
		"TAVR"[Title/Abstract] OR "TAVI"[Title/Abstract]
	)
	AND
	(
		"Pacemaking"[Title/Abstract] OR "Conduction disturbance"[Title/Abstract] OR
		"LBBB"[Title/Abstract] OR "RBBB"[All Fields] OR "Thrombocytopenia"[Title/Abstract] OR
```

```
        "platelet number"[Title/Abstract] OR "acute kidney injury"[Title/Abstract]
OR
        "AKI"[Title/Abstract] OR "paravalvular leak"[Title/Abstract] OR
        "mean pressure gradient"[Title/Abstract] OR "bicuspid valve"[Title/Abstract]
OR
        "horizontal aorta"[Title/Abstract] OR "aortic angulation"[Title/Abstract] OR
        "small annulus"[Title/Abstract] OR "endocarditis"[Title/Abstract] OR
        "coronary artery obstruction"[Title/Abstract] OR "commissural align-
ment"[Title/Abstract] OR
        "balloon valvuloplasty"[Title/Abstract] OR "cusp calcification"[Title/Abstract]
OR
        "left ventricular systolic dysfunction"[Title/Abstract] OR "sex difference"[Ti-
tle/Abstract]
    )
)
NOT
(
    "Transcarotid access"[Title/Abstract] OR "Transapical access"[Title/Abstract] OR
    "Substernal access"[Title/Abstract] OR "Transcaval access"[Title/Abstract] OR
    "Transaortic access"[Title/Abstract]
)
AND "humans"[MeSH Terms]
NOT
(
    "review"[Publication Type] OR "systematic review"[Publication Type] OR
    "meta-analysis"[Publication Type] OR "review"[Title] OR
    "systematic review"[Title] OR "meta-analysis"[Title] OR
    "meta analysis"[Title] OR "network meta-analysis"[Title/Abstract] OR
    "network meta analysis"[Title/Abstract]
)
AND
(
    "multicenter study"[Publication Type] OR "multicenter"[Title/Abstract] OR
    "multi-center"[Title/Abstract] OR "single-center"[Title/Abstract] OR
    "single centre"[Title/Abstract]
)
AND ("2020/01/01"[Date - Publication] : "2025/12/31"[Date - Publication]) AND "free
full text"[Filter]
```

Abbreviations: AKI, Acute Kidney Injury; LBBB, Left Bundle Branch Block; MeSH, Medical Subject Headings; RBBB, Right Bundle Branch Block; TAVI, Transcatheter Aortic Valve Implantation; TAVR, Transcatheter Aortic Valve Replacement.

Table S2. Study-level characteristics of the included studies.

Reference	Setting	Sample size (n)	Valve category / platform(s)	Comparison type	Main domain(s)	Follow-up / reporting	Key extracted findings
**Worthley et al. 2025 [37]**	36 sites across Europe, Australia, and Israel	**n=434 (low risk n=203; intermediate risk n=231)**	SEV (intra-annular THV); Navitor (Abbott)	Single-arm; no direct comparator	Safety/performance; PVL; hemodynamics in low-/intermediate-risk native severe AS	30-day and 12- month reported; annual follow-up planned to 10 years	434 patients (203 low risk, 231 intermediate risk); technical success 97.0%; no procedural mortality; moderate or greater PVL 0% at 30 d; primary 12-mo safety endpoint 2.3% in the first 262 cohort.
**Bunc et al. 2025 [38]**	Single-centre TAVI registry, University Medical Centre Ljubljana, Slovenia; October 2017-August 2023	**Registry n=1,152; comparison cohort n=144 (Myval n=97; Portico n=47); PSM n=68 (34 pairs)**	Intra-annular BEV vs intra-annular SEV; Meril Myval series BEV vs Abbott Portico series SEV; 3 Navitor cases in the Portico cohort	Direct head-to-head, non-randomized comparison; unmatched and propensity-matched cohorts	30-day and 1-year mortality, PPI, PVL, complications, and post-procedural hemodynamics	In-hospital/periprocedural outcomes plus 30-day and 1-year follow-up; routine pre-discharge echocardiography	All-comer TAVI registry, Oct 2017-Aug 2023. Myval n=97 vs Portico n=47; matched 34 vs 34. No significant between-group differences in 30-day/1-year mortality, new PPI, >mild PVL, or mean gradient.

**Gada et al. 2025 [31]**	11 centres, United States	**n=151**	SEV; Evolut FX (Medtronic)	Single-arm; no direct comparator	TAVR with cusp overlap technique (COT); procedural and CT-assessed commissural/ coronary alignment; PPI and AR outcomes	30-days and 1-year; median follow-up 371-days (Q1-Q3: 352-388 days)	151 patients with severe aortic stenosis received the device (September 2022-October 2023); refined 3-step COT compliance 86.0%; primary endpoint (all-cause mortality or all stroke) 2.7% at 30 days and 7.5% at 1 year; new PPI 6.7% at 30 days and 8.8% at 1 year; no moderate or greater total AR at discharge; 1/124 moderate total AR at 1 year; median length of stay 1 day.
**Kanehama et al. 2025 [76]**	Iwate Medical University, Japan (SC)	**n=339 (S3UR n=69; S3 n=270)**	BEV; SAPIEN 3 Ultra RESILIA (S3UR) vs SAPIEN 3 (S3)	Direct comparative cohort	Subclinical hemolysis and associated factors	Discharge and 1-month outcomes; primary hemolysis assessment at 1 month	339 TF-TAVI patients with severe aortic stenosis (S3UR 69; S3 270). Subclinical hemolysis at 1 month: 14.5% vs 2.7% (P<0.001). In S3UR, mild or greater 1-month PVL, lower annular oversizing, and higher SOV-THV were associated with hemolysis; SOV-THV remained significant in multivariable analysis.
**Bihag et al. 2025 [50]**	Hartford Hospital, Hartford, Connecticut, USA; transfemoral TAVI for native severe AS	**Original cohort n=2,461 (BEV n=1,685; SEV n=776); matched cohort n=1,363 (BEV n=683; SEV n=680)**	Contemporary/recent-generation BEV vs SEV; BEV: Sapien 3, Ultra; SEV: Evolut R, PRO, PRO+	Direct head-to-head BEV vs SEV; ViV/nontransfemoral excluded	Long-term clinical outcomes; hemodynamics/PPM; PPI; SLT/TIA	In-hospital, 30-day echo, intermediate echo subset, 1-year, median 77.0 months	Matched BEV 683 vs SEV 680. Similar in-hospital mortality/stroke, 1-year mortality/readmission/KCCQ12, and late all-cause mortality. BEV had lower PPI (8.3% vs 13.4%) but higher gradients, smaller EOA, more PPM, and more SLT; SEV had more TIA during follow-up.

**Hahn et al. 2025 [57]**	PARTNER 2 SAPIEN 3 intermediate-risk registry/single-arm study + PARTNER 3 low-risk RCT; TF-TAVR	**n=1,355 (small annulus n=476; large annulus n=879)**	BEV; SAPIEN 3	Small (≤430 mm²) vs large (>430 mm²) annuli within SAPIEN 3 cohort; small-annulus subgroups by 30-day MG and PPM	Small annulus; residual MG; PPM; late clinical outcomes and valve durability	1- and 5-year clinical, echocardiographic, KCCQ, BVF, and reintervention outcomes	n=1,355 (476 small; 879 large). 5-year primary endpoint similar (36.3% vs 35.8%; P=0.83). In small annuli, outcomes were not associated with 30-day MG or PPM; female subgroup also showed similar outcomes.
**Van Mieghem et al. 2025 [59]**	39 TAVI centres; 14 countries	**Enrolled n=638; TAVI attempted n=629**	SEV (supra-annular); Evolut PRO	Single-arm; no direct comparator	3-year outcomes; hemodynamics; PVL; PPI	Clinical to 3 - year; echocardiography at discharge, then in 1-year, then in 3-years	FORWARD PRO enrolled 638 patients; TAVI was attempted in 629. At 3 years, all-cause mortality was 25.0%, disabling stroke 6.5%, and new PPI 24.7%. Valve haemodynamics remained favourable (mean gradient 8.8 ± 4.7 mmHg; mean EOA 2.0 ± 0.5 cm²).
**Kini et al. 2025 [69]**	MC,United States; STS/ACC TVT Registry	**PSM: 4,598 matched pairs (S3UR n=4,598; S3/S3U n=4,598)**	BEV; SAPIEN 3 Ultra RESILIA (S3UR) vs SAPIEN 3 / SAPIEN 3 Ultra (S3/S3U)	Direct comparative, propensity-matched	1-year clinical and echocardiographic outcomes; PVL and hemodynamics	Discharge, 30 days, and 1 year; median follow-up 358 vs 364 days	Native TAVR; 4,598 matched pairs. S3UR was associated with lower 1-year all-cause mortality (7.6% vs 9.7%), lower life-threatening bleeding (2.0% vs 2.7%), lower mild-or-greater PVL at 1 year (15.6% vs 18.5%), larger EOA, and lower gradients; valve reintervention was rare.

**Van Royen et al. 2025 [39]**	MC; 31 centres in 16 countries	**n=768 (Myval n=384; SAPIEN n=192; Evolut n=192)**	BEV vs BEV and BEV vs SEV; Myval/Myval Octacor; SAPIEN 3/SAPIEN 3 Ultra; Evolut R/PRO/PRO+/FX	Direct randomised head-to-head 3-arm comparison	Early clinical safety/effectiveness and hemodynamic performance	30-day clinical and echocardiographic outcomes	Adults with severe symptomatic native aortic stenosis; n=768 (Myval 384, SAPIEN 192, Evolut 192). Myval was non-inferior to both SAPIEN and Evolut for the primary composite endpoint. PPI rates were similar across arms (15.0%, 17.3%, 16.8%). Myval showed better hemodynamics vs SAPIEN, whereas Evolut showed superior hemodynamics vs Myval. Moderate-severe prosthetic valve regurgitation was numerically higher with Evolut (7.4%) vs Myval (3.4%).
**Rück et al. 2025 [63]**	International MC; 10 European centres	**n=181; analysable MSCT n=160; post-TAVI core-lab echocardiography n=164**	SEV; ACURATE neo2 (Boston Scientific)	Single-arm	Safety/efficacy and valve performance in BAV	30-day VARC-3 clinical + echocardiography	n=181; core-lab imaging; technical success 95.6%; device success 90.6%; early safety 82.3%; stroke 1.6%; PPI 6.5%; moderate PVL 4.3%
**Nakase et al. 2025 [51]**	SC; Bern University Hospital, Switzerland (Bern TAVI registry / SwissTAVI)	**n=759; PSM n=268 (134 pairs)**	BEV vs SEV THV in patients with reduced LVEF undergoing TAVI; BEV: SAPIEN XT, Sapien 3, Sapien 3 Ultra; SEV: CoreValve, Evolut R/PRO/PRO Plus	Direct comparative; 1:1 propensity-score-matched BEV vs SEV cohort	Severe AS with LV systolic dysfunction (LVEF <50%): procedural safety, hemodynamics/PVR, and long-term outcomes	Discharge echocardiography; 30-day, 1-year, and 5-year follow-up	759 patients with LVEF <50%; PSM yielded 134 pairs. SEV was associated with a lower discharge mean gradient (7.1 ± 3.7 vs 9.9 ± 4.3 mmHg) and more ≥mild-to-moderate paravalvular regurgitation (36.3% vs 11.3%). At 5 years, observational adjusted analysis showed higher all-cause mortality with SEV (67.8% vs 55.8%; adjusted HR 1.44, 95% CI 1.02-2.03).

**Feistritzer et al. 2025 [49]**	7 German sites; intermediate- to high-risk patients with severe, symptomatic aortic stenosis undergoing transfemoral TAVR	**n=447 randomized**	Newer-generation SEV vs BEV; Evolut R (Medtronic) vs SAPIEN 3 (Edwards Lifesciences)	Direct head-to-head randomized comparison	5-year valve-related clinical outcomes; concurrent anesthesia strategy analysis	5-year outcomes	n=447 randomized. Predefined valve composite endpoint was similar between SEV and BEV (67.7% vs 63.4%; HR 0.89, 95% CI 0.70-1.13; P=0.34). Stroke was lower with SEV (2.2% vs 9.6%; HR 4.84, 95% CI 1.65-14.18; P=0.002). Mean 5-year gradient was lower with SEV in available echocardiographic follow-up (7 vs 11 mm Hg; P=0.005).
**Modine et al. 2024 [77]**	International MC (86 centres)	**Age <75 y subgroup n=703 (TAVR n=352; surgery n=351)**	Supra-annular SEV; Medtronic CoreValve / Evolut R / Evolut PRO; surgical bioprosthetic valve model NR	SEV TAVR vs surgery	3-year clinical outcomes, bioprosthetic valve performance, and quality of life in younger (<75 years) low-surgical-risk severe aortic stenosis	3-year main report; 30-day and 1-year outcomes also reported; median follow-up 48.7 months (TAVR) and 48.1 months (surgery)	703 patients <75 years (352 TAVR, 351 surgery); disabling stroke lower with TAVR (0.6% vs 2.9%), but PPI higher (21.0% vs 7.1%); lower gradients and less severe PPM with TAVR; mortality, reintervention, thrombosis, and endocarditis were similar; >=moderate PVL not significantly different.
**Chen et al. 2024 [54]**	PARTNER 2 S3 registries (S3i/S3HR)	**n=857 without a prior permanent pacemaker**	BEV; SAPIEN 3	No direct BEV-SEV comparison; new PPI vs no new PPI	Permanent pacemaker implantation after TAVR: incidence, predictors, and 5-year outcomes	New PPM within 30 days; 5-year clinical/echocardiographic follow-up (median 1,682 days)	857 patients without a prior permanent pacemaker; 12.5% underwent new PPI. Independent predictors: older age, baseline RBBB, moderate-to-severe annular calcification, valve oversizing, 26/29-mm prosthesis, and implant depth >6 mm. No significant increase in 5-year all-cause mortality or rehospitalization; LVEF remained lower during follow-up.

**Frank et al. 2024 [78]**	28 centres in 7 European countries	**Pre-implementation n=897; post-implementation n=1,491**	BEV; SAPIEN 3; SAPIEN 3 Ultra	Before-after BENCHMARK implementation; no direct BEV-SEV comparison	Streamlined TAVI pathway; LoS; critical care use; procedural efficiency; safety	In-hospital/discharge and 30-day reported; 12-month follow-up planned	Retrospective pre-implementation cohort vs prospective post-implementation cohort; n=897 vs 1491; 8 best practices; mean LoS 7.7 to 5.8 days; critical care stay 1.8 to 1.3 days; QoL assessed only post-implementation
**Costa et al. 2024 [58]**	SC; Catania, Italy	**n=127; post-TAVR CTA n=115**	SEV (supra-annular); Evolut R/PRO/PRO+; ACURATE neo2	Direct SEV platform comparison	Coronary reaccess/cannulation after TAVR; commissural alignment and CTA predictors	Immediate post-TAVR angiography; post-TAVR CTA in 115/127 (90.6%)	127 patients; unsuccessful coronary cannulation 5.5% overall, mostly Evolut (6/7). Severe misalignment predicted failure of both coronaries; RCA overlap predicted unsuccessful selective RCA cannulation.
**Mas-Peiro et al. 2024 [79]**	4 high-volume TAVR centres (Canada and Europe)	**n=740**	SEV (supra-annular); Evolut R / Evolut PRO / Evolut PRO+ (Medtronic)	Internal oversizing comparison (quartiles; <20% vs ≥20%)	Valve oversizing and moderate-to-severe PVL; post-dilation; PPI	Procedural, discharge, and in-hospital outcomes	740 consecutive Evolut recipients with CT data. Moderate-to-severe PVL occurred in 7.0%. Greater oversizing was associated with lower PVL and less post-dilation; a 20% oversizing threshold best discriminated PVL in this observational analysis. PPI was similar below vs at/above the 20% threshold (18.4% vs 18.3%).
**Elkoumy et al. 2024 [70]**	Europe; severe native AS; transfemoral TAVI; Sept 2020–Feb 2023	**n=499 (balloon predilatation n=391; direct TAVI n=108); PSM n=168 (84 pairs)**	SEV; ACURATE neo2	Balloon predilatation vs direct TAVI within the same SEV platform	Predilatation strategy; anatomy/calcification; procedural and early outcomes	Procedural, in-hospital, and predischarge reporting	n=499 (balloon predilatation n=391; direct TAVI n=108); PSM 84 pairs. Direct-TAVI patients had smaller annuli and less AV calcification. Matched technical success was 95% in both groups; early outcomes were comparable.

**Briedis et al. 2023 [41]**	SC, Kaunas, Lithuania	**n=10**	SEV; Vienna Aortic Valve Self-Expandable System	Single-arm; no direct comparator	Early safety, feasibility, hemodynamics, and quality of life	Procedural/discharge assessments and 30-day outcomes	Premounted dry-pericardium supra-annular SEV; repositionable/retrievable; n=10 symptomatic severe aortic stenosis; technical and device success 100%; 30-day all-cause mortality 0%; stroke 2/10; new PPI 2/9 (22.2%); moderate PVL 1/10.
**Zaid et al. 2023 [32]**	MC, United States (9 centres)	**Evolut FX n=226; historical Evolut PRO+ comparator n=378**	SEV (supra-annular); Medtronic Evolut FX; comparator analysis with Evolut PRO+	Historical single-centre within-platform comparison vs Evolut PRO+ (n=378)	Commissural alignment, implant depth, device recapture, PVL, and 30-day clinical/echocardiographic outcomes	In-hospital and 30-day reporting	226 consecutive transfemoral TAVR patients. Commissural alignment was 96.5% and technical success 99.1%. Compared with Evolut PRO+, FX showed fewer recaptures/repositioning (26.1% vs 39.5%), better alignment (96.5% vs 80.2%), and shallower LCC depth (4.5 ± 2.1 vs 5.9 ± 2.2 mm). At 30 days: mortality 1.3%, stroke 1.3%, PPI 11.9%, mild PVL 14.3%, moderate PVL 0.9%, severe PVL 0%.
**Chandrasekar et al. 2023 [55]**	SC; Kuwait	**n=190**	BEV; Sapien 3	Single-arm cohort; internal subgroup comparison	PPI risk; infra-annular extension of native RCC/NCC	Post-TAVI PPI reporting; duration NR	n=190; transfemoral only. Infra-annular RCC/NCC extension in 17.37%; PPI 36.36% vs 8.92% without extension (RR 4.08). Preexisting RBBB and infra-annular extension were independent predictors of PPI.
**Nikolayevska et al. 2024 [66]**	ViV-TAVI in symptomatic degenerated SAV; 2008-2018; n=112	**n=112**	ViV in failed surgical bioprostheses; SEV (supra-annular) vs BEV; ALLEGRA; CoreValve/Evolut R; SAPIEN/SAPIEN XT/SAPIEN 3	3-arm direct comparison	Hemodynamics and procedural safety	Discharge/in-hospital only; no long-term follow-up	Procedural success 94.6%; 58.9% had true ID <21 mm. Mean gradient decreased from 33.7±16.5 to 18.0±7.1 mmHg. ALLEGRA vs CoreValve/Evolut R: 14.9±5.0 vs 18.7±7.5 mmHg (p=0.0295); complication rates were similar.

**Reardon et al. 2023 [72]**	26 clinical sites across Europe, Australia, and the United States	**n=260**	SEV; intra-annular THV; Navitor TAVR System (Abbott Structural Heart); FlexNav delivery system	Single-arm, non-comparative	30-day safety, PVL, hemodynamics, and PPI	30 days reported; follow-up planned at 1 year and annually through 5 years	Symptomatic severe native AS at high/extreme surgical risk; n=260; 0% moderate or greater PVL; all-cause mortality 1.9%; new PPI 19.0% in pacemaker-naive subjects; mean gradient 7.4 ± 3.5 mm Hg; EOA 2.00 ± 0.47 cm²
**Leone et al. 2023 [52]**	International; 16 high-volume centres; June 2011-April 2020	**n=1,378 (SEV n=1,092; BEV n=286; supra-annular n=920; intra-annular n=458)**	Small annuli; SAV vs IAV; SEV vs BEV; Evolut R/PRO; ACURATE neo; Portico; SAPIEN 3	Direct comparative, non-randomised; IPTW-adjusted	Hemodynamics, PPM, PVL, PPI, clinical outcomes	Predischarge outcomes; median follow-up 377 days (IQR 168-700)	1,378 patients with severe native aortic stenosis and small annuli treated transfemorally. SAV/SEV had lower mean gradients and lower severe PPM than IAV/BEV; more than mild PVL was higher with SEV vs BEV; no all-cause mortality difference.
**Sondergaard et al. 2023 [36]**	19-centre study; severe symptomatic AS; high/extreme surgical risk	**n=120**	SEV; Navitor THV	Single-arm; no direct comparator	Safety/performance, PVL, hemodynamics, PPI, functional status/QoL	30 days and 1 year; annual follow-up to 5 years ongoing	CE-mark cohort (n=120); procedural success 97.5%; mortality 0% at 30 days and 4.2% at 1 year; moderate/severe PVL 0% at 30 days and moderate PVL 1.0% at 1 year; new PPI 15.0% at 30 days.
**Toggweiler et al. 2022 [80]**	4 centres; Switzerland, Canada, Argentina	**n=157 (ACURATE neo n=100; ACURATE neo2 n=57)**	SEV-only; Acurate neo; Acurate neo2	Single-arm	Single femoral access ("all-in-one")	In-hospital + 30-day	n=157; all procedures successful via a single large-bore sheath; discharge mean gradient 7 ± 3 mm Hg; >mild PVL 7/157; 30-day major vascular complications 2/157, stroke 2/157, new PPI 4/157, mortality 3/157.

**Buono et al. 2022 [33]**	13 Italian high-volume centres; patients with severe native aortic stenosis undergoing TAVR.	**Registry n=900 (neo2 n=220; neo n=680); PSM n=410 (205 pairs)**	SEV; ACURATE neo and ACURATE neo2 (Boston Scientific)	Direct first- vs second-generation within-family comparison.	Postprocedural hemodynamics and short-term clinical outcomes, especially paravalvular AR/PVL, technical success, device success, and safety.	Predischarge echocardiography and in-hospital outcomes; 90-day clinical and echocardiographic follow-up.	Registry n=900 (ACURATE neo2 n=220; ACURATE neo n=680); 1:1 propensity-matched cohort n=205 vs 205. Moderate-or-greater paravalvular AR was lower with ACURATE neo2 at predischarge (3.5% vs 11.2%, P<0.001) and at 90 days (3.5% vs 12.7%, P=0.002); technical success was similar (97.6% vs 95.1%, P=0.293).
**Kim et al. 2022 [81]**	SC, Germany	**n=1,417 (postdilatation n=521; no postdilatation n=896)**	SEV; Acurate neo/neo2	Balloon postdilation vs no postdilation	Postdilation efficacy and safety; paravalvular regurgitation, PPM, and predictors	Intraprocedural, discharge, and 30-day reporting	n=1417; balloon postdilation in 521 (36.8%), mainly for relevant paravalvular regurgitation; success 87.9%; >mild paravalvular regurgitation fell from 32.0% to 7.5%; no excess major stroke, acute kidney injury, PPI, or 30-day mortality; THV damage 1.2%.
**Loewenstein et al. 2022 [82]**	MC; Israeli national TAVR registry (4 centres, Israel)	**Registry n=2,591; propensity-matched cohorts: Evolut PRO n=222, CoreValve n=212, Evolut R n=213**	SEV (generational); Medtronic CoreValve, Evolut R, Evolut Pro	Direct comparative EPV vs CV; EPV vs ERV	PVL, permanent pacemaker implantation, procedural safety/efficacy	Postprocedural outcomes; echocardiography closest to 1 month when available	Israeli registry n=2,591; propensity-matched cohorts included Evolut PRO n=222, CoreValve n=212, and Evolut R n=213. Moderate-or-greater PVL was lower with Evolut PRO than CoreValve but not significantly different from Evolut R; PPI was lower with Evolut PRO (16.3%) than CoreValve (33.5%) and Evolut R (24.4%).

**Deeb et al. 2022 [61]**	25 U.S. sites; low-surgical-risk severe symptomatic AS or class IIa asymptomatic indication	**Bicuspid cohort n=150 attempted TAVR; PSM 145 pairs**	SEV; supra-annular THV; Evolut R or Evolut PRO (Medtronic)	Propensity-matched bicuspid vs tricuspid comparison	1-year procedural, clinical, hemodynamic, PVL, PPM, and QoL outcomes	30 days and 1 year	Bicuspid cohort n = 150 attempted TAVR; 145 matched pairs; device success 95.8% vs 96.5%; 1-year all-cause mortality or disabling stroke 1.4% vs 2.8%; new PPI 17.1% vs 18.2%; EOA 2.2 vs 2.3 cm2; mean gradient 8.7 vs 8.5 mm Hg; mild-or-worse PVL 21.3% vs 42.6%.
**Aidietis et al. 2022 [40]**	International; 18 centres in Europe and Asia-Pacific	**n=157**	SEV; Hydra THV	Single-arm; no comparator	Safety/performance; EOA, gradient, PVL, PPI	30-day and 1-year outcomes	Symptomatic severe AS at high/extreme surgical risk; N=157; successful implantation 94.3%; 30-day mortality 7.0%; 1-year mortality 14.6%
**Voigtländer et al. 2021 [56]**	4 high-volume centres, Germany	**Small-annulus analytic cohort n=1,069 (parent cohort n=8,411)**	Small aortic annulus; supra-/intra-/infra-annular THV; Sapien 3; Evolut/Evolut R/Pro; Acurate Neo; Portico; Lotus	5-group device comparison	PPM / hemodynamics / PVL	Discharge echo; 30-day and 12-month outcomes	Transfemoral TAVI; annulus area <400 mm^2. n=1069/8411 (12.7%). Overall PPM 38.3% (28.1% moderate; 10.2% severe). SE THV had lower PPM than Sapien 3/Lotus, but >mild PVL was more frequent. 30-day and 12-month mortality were similar.
**Van Mieghem et al. 2022 [60]**	Routine clinical practice; 53 centres in 20 countries; symptomatic severe native aortic stenosis or failed surgical bioprosthesis	**n=1,039; valve-in-valve subgroup n=50**	SEV, supra-annular, repositionable THV; Evolut R (Medtronic)	Single-arm; within-cohort subgroup analyses	3-year safety/clinical outcomes; PPI, paravalvular regurgitation, THV-related complications, ViV feasibility	Clinical follow-up to 3 years; echocardiography to 1 year; n = 1039	All-cause mortality 24.8% and disabling stroke 4.8% at 3 years. New PPI and more than trace paravalvular regurgitation were not associated with mortality. Low repeat intervention (1.1%), valve thrombosis (0.2%), and endocarditis (0.9%); ViV subgroup n = 50.

**Möllmann et al. 2021 [34]**	9 European centres	**n=120**	SEV; ACURATE neo2	None (single-arm)	Clinical safety, hemodynamics, PVL and PPI after TAVI	30 days and 1 year	n=120; procedural success 97.5%; mortality 3.3% at 30 days and 11.9% at 1 year; new PPI 15.0% at 30 days and 17.8% at 1 year; 1-year PVL: 2.5% moderate, 0% severe.
**Feistritzer et al. 2021 [83]**	MC; 7 sites; Germany	**n=447 randomized**	Newer-generation SEV vs BEV; Evolut R (SEV) vs Sapien 3 (BEV)	Head-to-head randomized comparison; GA vs CS arm	1-year clinical outcomes after transfemoral TAVR	1 year	447 intermediate- to high-risk severe symptomatic aortic stenosis patients; composite endpoint similar between SEV and BEV; stroke higher with BEV; SEV had lower valve gradients; GA and CS showed similar outcomes.
**Schaefer et al. 2021 [84]**	SC; Hamburg, Germany	**n=79**	BEV; SAPIEN 3 Ultra	None; single-arm	30-day outcomes; preprocedural CT predictors of PPI/PVL	30-days; VARC-2	n=79 consecutive transfemoral TAVI; device success 97.5%; early safety 94.9%; mortality 2.5%; PPI 7.6%; mild PVL 18.9%; PVL > mild 0%; higher right-coronary cusp calcium associated with PPI; higher eccentricity index associated with mild PVL.
**Modolo et al. 2021 [85]**	Amsterdam UMC, Netherlands; SC consecutive TAVR cohort	**n=100**	BEV; Edwards SAPIEN 3; SAPIEN 3 Ultra	Online vs blinded offline core-lab analysis	Post-TAVR AR assessment; online feasibility/reproducibility	Intraprocedural assessment; repeat image analysis at 30-days	100 patients; SAPIEN 3 97%, SAPIEN 3 Ultra 3%. Online analyzability 92%, identical to core lab; ICC 0.962. Operators did not use online values for implantation optimization.
**Yamada et al. 2020 [67]**	Japan; transfemoral TAVI; March 2014-August 2019	**n=131 (platelet decrease >=50% n=74; <50% n=57)**	BEV vs SEV; Edwards Sapien XT, Sapien 3; Medtronic CoreValve, EvoluteR, Evolute-Pro	DPC >=50% vs <50%	Predictors of short-term thrombocytopenia / DPC after TAVI	Platelets measured pre-TAVI, day 0, 1, 2, 3, 5, 7, then as needed; nadir up to 14-days	n=131; 74 with DPC >=50%, 57 with DPC <50%; nadir on day 3; low BMI and BEV independently predicted DPC >=50%

**Fontana et al. 2020 [35]**	28 sites in the United States, Australia, and Europe; high-/extreme-risk symptomatic severe native AS; attempted transfemoral implantation	**n=180**	SEV; intra-annular THV; Portico THV with FlexNav delivery system	Non-comparative pooled single-arm study	Safety profile of next-generation low-profile delivery system; major vascular complications	30-day outcomes only	N = 180; technical device success 96.7%; major vascular complications 5.0% (4.4% access site-related; 3.3% delivery-system access site-related); all-cause mortality 0.6%; disabling stroke 1.1%; new PPI 15.4%; moderate PVL 4.1%
**Kaneko et al. 2020 [65]**	SC; Sapporo, Japan	**n=7**	SEV; Evolut R / Evolut PRO	Single-arm	Extremely horizontal aorta (aortic root angle >=70 degrees); technical aspects and clinical outcomes	Echo at day 1 and 30; VARC-2 outcomes and mortality at 3 months	n=7; 100% device success; no post-dilatation; PVL no more than mild; 2 modified implantation techniques; complications: PPI, stroke, tamponade
**Armijo et al. 2020 [53]**	12 centres; Europe/North America	**n=833 (SAPIEN 3 n=640; Evolut R n=193); extra-large annulus subgroup n=124 (n=95; n=29)**	BEV vs SEV; 29-mm Sapien 3; 34-mm Evolut R	Head-to-head	Large/extra-large annuli; device success, PVL, hemodynamics, clinical outcomes	Predischarge/ in-hospital, 30-day, 1-year	n=833 (S3 640; ER 193). S3 had higher device success (95.8% vs 89.3%) and lower moderate-severe AR (1.6% vs 5.9%); mortality similar. Extra-large subgroup (n=124): similar device success (94.6% vs 93.1%).

**Manoharan et al. 2020 [86]**	International; 39 centres in 14 countries	**Enrolled n=638; TAVI attempted n=629; implanted n=610**	SEV; Evolut PRO	Single-arm; prespecified performance-goal comparison	30-day safety, clinical outcomes, hemodynamics, and AR/PVL	Discharge and 30 days; 5-year follow-up planned	629 attempted (610 implanted). 30-day all-cause mortality 3.2% vs 5.5% goal; disabling stroke 2.9%; major vascular complication 3.0%; moderate/severe AR 1.8%; new PPI 20.7%; no/trace AR endpoint not met.
**Chow et al. 2020 [87]**	SC; Prince of Wales Hospital, Hong Kong	**n=88 total; PPI analysis n=85 (3 patients with pre-existing pacemakers excluded)**	SEV; CoreValve Evolut R; Portico; Hydra	Single-arm / no direct comparator	Short-term clinical outcomes and risk factors for PPI after self-expandable TAVI	30-day and 1-year outcomes; 1-year pacemaker dependence assessment	88 patients. 30-day PPI 17.6% (15/85; 3 pre-op pacemaker cases excluded). Pre-TAVI RBBB, deeper NCC implantation depth, and NCC depth/IMS > 0.5 were associated with higher PPI risk. 40% of patients with post-procedural pacemaker implantation were non-pacemaker-dependent at 1 year.
**Modolo et al. 2020 [88]**	MC, multicontinental, real-world cohort	**TAVR cases evaluated n=3,976; analyzable aortograms n=2,258**	Multiple commercially available THV platforms; ACURATE; Centera; CoreValve; Direct Flow Medical; Evolut PRO; Evolut R; Inovare; Lotus; Lotus Edge; SAPIEN XT; SAPIEN 3	Non-randomized multi-valve comparison	Acute post-TAVR regurgitation / sealing performance assessed by videodensitometry	Immediate acute post-implantation; final aortogram only	3,976 TAVR cases evaluated; 2,258 analyzable aortograms by an independent academic core laboratory using video densitometry. Main analysis included 7 valve types with >50 procedures. Lotus had the lowest mean LVOT-AR (3.5 ± 4.4%) and moderate/severe regurgitation (2.2%); first-generation CoreValve had the highest (13.7 ± 10.7%; 30.1%).

**Owais et al. 2020 [68]**	SC; Zentralklinik Bad Berka, Germany	**n=98 (no contrast n=25; contrast n=73)**	BEV; Edwards Sapien balloon-expandable bioprostheses (Sapien 3)	TF-TAVR without contrast medium vs TF-TAVR with contrast medium	Contrast-free TF-TAVR in severe nephropathy; acute kidney injury and procedural outcomes	In-hospital and 30-day follow-up	98 consecutive patients (25 without contrast medium; 73 with contrast medium); GFR <30 mL/min/1.73 m2; most patients received a 26 mm Edwards Sapien 3 valve; acute kidney injury 4.0% vs 20.5% (P=.04); no in-hospital mortality; 30-day mortality 4.0% vs 2.7%; no periprocedural stroke or major vascular complications.
**Waksman et al. 2020 [62]**	MC, 6 US centres; low-risk symptomatic severe bicuspid AS	**n=61**	BEV + SEV; SAPIEN 3; Evolut R/Evolut PRO	Single-arm bicuspid TAVR; no direct BEV vs SEV comparison	30-day safety, hemodynamics, PVL, PPI, and HALT	30-day primary endpoint/reporting; baseline/discharge/30-day echocardiography; 30-day CT	n=61; mean age 68.6 years; 100% transfemoral; 74% BEV/26% SEV; 30-day mortality 0%, disabling stroke 0%, new PPI 13.1%, >mild PVL 1.6%, HALT 10.2%; Sievers type 1 78.3%
**Duran Karaduman et al. 2020 [89]**	Tertiary care centre, Turkey	**n=556**	Mixed THV cohort; mostly BEV; Sapien XT; Sapien 3; Lotus; ACURATE neo	Descriptive THV model comparison	Procedural and clinical outcomes; PPI, PVL, mortality	In-hospital, 30-day, 1-year; mean follow-up 15.1±14.9 months	556 consecutive patients. BEV 94.6%; device success 96.6%. PPI 7.2% overall, highest with Lotus (37.5%). PVL 18.9% at discharge; Sapien 3 had no moderate PVL.

**Tang et al. 2020 [30]**	5-centre TAVR cohort for aortic stenosis; pre-TAVR CT plus procedural fluoroscopy; March 2016-September 2019	**n=828 (SAPIEN 3 n=483; Evolut n=245; ACURATE neo n=100)**	Multiplatform THV; SAPIEN 3; Evolut; ACURATE-neo	Within-platform initial orientation comparison	Commissural alignment and coronary overlap	Periprocedural imaging only; no systematic clinical outcomes	n=828 (483/245/100); severe overlap = 0 to 20 degrees. Initial SAPIEN 3 orientation had no impact. Evolut OC/CF and ACURATE-neo CB/IC were linked to better alignment and less overlap. Intentional ACURATE-neo alignment succeeded in 5/7 cases.
**Rogers et al. 2020 [29]**	U.S.; MC low-risk TAVR cohort	**LRT parent cohort n=200; SAPIEN 3 n=177; 30-day CT n=168; analyzable CT n=137**	BEV; Edwards SAPIEN 3	Single-arm BEV analysis; intentional vs random crimping subgroup	Coronary access; commissural alignment; TAVR-in-TAVR feasibility	30-day post-TAVR CT; in-hospital outcomes	177 SAPIEN 3; 168 CT; 137 analyzable. High-risk coronary access anatomy 9-13%; intentional crimping no meaningful benefit; frame above STJ with VTSTJ <2 mm in 13.1%.
**Sengupta et al. 2020 [64]**	15 centres in North America	**n=105**	BEV; Edwards SAPIEN 3 (29-mm)	Single-arm; no direct comparator	Extremely large annuli (>683 mm^2); mid-term clinical and echocardiographic outcomes	In-hospital, 30-day, and 1-year outcomes; VARC-2 reporting; 1-year survival follow-up 94.3%; 1-year echocardiographic follow-up 82.1%	105 patients with symptomatic severe aortic stenosis; mean annular area 721.3 ± 36.1 mm^2; procedural success 100%; 1-year mortality 18.2%; stroke 2.4%; mild PVL 21.7%; moderate/severe PVL 4.3%

Abbreviations: ACC, American College of Cardiology; AR, aortic regurgitation; AS, aortic stenosis; AV, aortic valve; BAV, bicuspid aortic valve; BEV, balloon-expandable valve; BMI, body mass index; BVF, bioprosthetic valve fracture; CB, center back; CE, European Conformity; CF, center front; CI, confidence interval; COT, cusp overlap technique; CS, conscious sedation; CT, computed

tomography; CTA, computed tomography angiography; CV, CoreValve; DPC, decrease in platelet count; EOA, effective orifice area; EPV, Evolut PRO valve; ER, Evolut R; ERV, Evolut R valve; GA, general anaesthesia; GFR, glomerular filtration rate; HALT, hypoattenuated leaflet thickening; HR, hazard ratio; IC, inner curve; ICC, intraclass correlation coefficient; ID, internal diameter; IPTW, inverse probability of treatment weighting; IQR, interquartile range; KCCQ, Kansas City Cardiomyopathy Questionnaire; KCCQ-12, Kansas City Cardiomyopathy Questionnaire-12; LCC, left coronary cusp; LRT, Low Risk TAVR; LV, left ventricle; LVEF, left ventricular ejection fraction; LVOT, left ventricular outflow tract; MC, multicentre; MG, mean gradient; MSCT, multislice computed tomography; NCC, non-coronary cusp; NR, not reported; OC, outer curve; PPI, permanent pacemaker implantation; PPM, prosthesis-patient mismatch; PSM, propensity-score matching; PVL, paravalvular leak; Q1–Q3, first to third quartile; QoL, quality of life; RBBB, right bundle branch block; RCA, right coronary artery; RCC, right coronary cusp; RCT, randomized controlled trial; RR, relative risk; S3, SAPIEN 3; S3U, SAPIEN 3 Ultra; S3UR, SAPIEN 3 Ultra RESILIA; SC, single-centre; SE, self-expanding; SEV, self-expanding valve; SLT, subclinical leaflet thrombosis; SOV, sinus of Valsalva; STJ, sinotubular junction; STS, Society of Thoracic Surgeons; TAVI, transcatheter aortic valve implantation; TAVR, transcatheter aortic valve replacement; TF-TAVI, transfemoral transcatheter aortic valve implantation; THV, transcatheter heart valve; TIA, transient ischaemic attack; TVT, Transcatheter Valve Therapy; VARC, Valve Academic Research Consortium; ViV, valve-in-valve; VTSTJ, valve-to-sinotubular junction distance.

Revision note: The former Table 4 was transferred to the Supplementary Materials as Supplementary Table S2 and comprehensively reformatted to improve readability and participant-level transparency. A dedicated “Sample size (n)” column was added for all 50 included reports. Participant numbers were checked study-by-study against the primary reports and the manuscript, and overall, randomized/matched, imaging, and analyzable cohort sizes are distinguished where applicable. No missing denominator was estimated. To accommodate the additional sample-size information without increasing table complexity, the previous “Valve category/focus” and “Valve family/model explicitly reported” columns were consolidated into a single “Valve category/platform(s)” column. Terminology and abbreviations were also standardized throughout the table, including the use of PPI for permanent pacemaker implantation and PPM for prosthesis-patient mismatch.

Table S3. Deviations from and methodological refinements to the original thesis protocol

Protocol component	Original thesis protocol	Approach used in the completed review	Explanation
**Review design and quantitative synthesis**	The project was initially described as a systematic review and meta-analysis, with quantitative synthesis planned if feasible.	The review was completed as a systematic review without meta-analysis. Findings were synthesized narratively according to valve platform and clinical, procedural, or anatomical domain.	Meta-analysis was considered inappropriate because of substantial clinical and methodological heterogeneity in study designs, patient populations, valve platforms and generations, outcome definitions, follow-up durations, and completeness of reporting.
**Information sources**	PubMed was specified as the principal database. Reference lists of included studies were also planned to be examined for additional potentially eligible reports.	Study identification was restricted to the final PubMed search. Reference-list screening and searching of additional databases or registers were not undertaken.	Reference-list screening was not completed during the review workflow. No records from additional information sources were included. The restriction to a single database was acknowledged as a limitation of the review.
**Search-strategy operationalization**	Broad combinations of balloon-expandable and self-expandable valve terms with prespecified clinical and anatomical keywords were proposed.	The preliminary search framework was converted into a reproducible Boolean PubMed strategy containing device-platform, TAVI/TAVR procedure, and clinical or anatomical outcome blocks, together with field tags and relevant search restrictions.	The preliminary strategy was operationalized in greater detail to permit reproducible execution of the final database search and to improve the specificity of study identification.
**Publication period**	Studies published during approximately the preceding five years and not before 1 January 2020 were planned for inclusion.	A fixed publication window of 1 January 2020 through 31 December 2025 was applied. The final search was conducted on 25 January 2026.	The original contemporary five-year criterion was converted into a fixed calendar period to improve clarity and reproducibility.
**Regulatory-status criterion**	The protocol proposed the inclusion of TAVI devices described as CE- and FDA-approved.	Regulatory approval status was not applied as an inclusion or exclusion criterion in the completed review. No study was excluded solely because a valve lacked CE marking or FDA authorization.	The planned regulatory-status criterion was not operationalized because regulatory status was jurisdiction- and time-dependent, making consistent application difficult and potentially leading to arbitrary exclusions. Eligibility was therefore based on clinical relevance and the remaining prespecified methodological criteria.
**Discontinued-platform criterion**	The protocol proposed excluding valves that had been discontinued from the	Commercial market status may change during or after a prespecified review period. ACURATE neo/neo2 was commercially	Market status may change after study conduct or publication. Retrospective exclusion based on subsequent commercial

Protocol component	Original thesis protocol	Approach used in the completed review	Explanation
	market during the preceding five years.	available and clinically relevant during a substantial portion of the 2020–2025 evidence window and generated eligible contemporary clinical evidence before its commercial discontinuation in 2025. Retrospectively excluding these studies solely because the platform was subsequently discontinued would remove evidence that was relevant during the period under review. Accordingly, such studies were retained for completeness and historical/mechanistic interpretation, while evidence from discontinued platforms was not used to formulate recommendations for currently available prostheses. This represents a transparent deviation from the original thesis protocol.	discontinuation could therefore remove otherwise eligible evidence according to events occurring after publication. This represents a deviation from the original thesis protocol and is reported transparently.
**Access-route eligibility**	The protocol did not define exclusions according to the TAVI access route.	Reports focused on non-target access routes, including transcarotid, transapical, substernal, transcaval, and transaortic access, were excluded.	This restriction was introduced during operationalization of the eligibility criteria to maintain a more clinically coherent review scope and reduce heterogeneity related to substantially different access approaches.
**Eligible study designs and exclusion criteria**	The protocol broadly specified PubMed-indexed, freely accessible studies of contemporary balloon-expandable and self-expandable devices and excluded mechanical or discontinued valves.	The completed review explicitly included randomized controlled trials, comparative observational studies, registry analyses, prospective and retrospective cohorts, post hoc analyses with extractable data, and feasibility or first-in-human studies. It excluded case reports, reviews, editorials or letters without primary data, protocol-only reports, animal or preclinical studies, SAVR-only studies, mechanically expandable valves, and mixed-platform reports without extractable relevant data.	The eligibility criteria were specified in greater detail to support consistent, reproducible study selection and to distinguish eligible primary clinical evidence from non-eligible publication types.
**Risk-of-bias assessment**	A quality assessment was contemplated, particularly if meta-analysis was performed, but no specific	Risk of bias was assessed for all included studies using RoB 2 for randomized studies and	Design-specific validated instruments were introduced to strengthen the methodological appraisal and interpretation of

Protocol component	Original thesis protocol	Approach used in the completed review	Explanation
	appraisal instrument was identified.	MINORS for non-randomized studies.	the evidence despite the decision not to conduct meta-analysis.
**Study-selection procedures**	Sequential screening by title, abstract, and full text was planned, but reviewer roles and disagreement-resolution procedures were not specified.	Title and abstract screening was performed by I.A. Full-text eligibility decisions were assessed by I.A. and independently verified by G.P.G. Uncertainties were resolved through discussion, with K.L. consulted when necessary. No automation tools were used.	Reviewer roles, verification procedures, and disagreement resolution were defined more explicitly to improve methodological transparency and reduce the risk of selection errors.
**Data-extraction procedures**	The protocol identified the principal evidence domains but did not describe a standardized extraction form, verification process, or procedures for handling unclear information.	Data were extracted by I.A. using a structured evidence-extraction form and subsequently verified and curated by G.P.G. for accuracy, completeness, and consistency. Discrepancies were resolved through discussion, and unclear or unavailable information was recorded as not reported.	Structured extraction and verification procedures were introduced to improve consistency, accuracy, and transparency.
**Outcome and follow-up data handling**	The protocol prespecified the principal clinical, procedural, and anatomical domains but did not provide detailed rules for handling follow-up time points or multiple estimates.	The core prespecified domains were retained and supplemented by standard clinical and valve-performance outcomes reported by eligible studies. Relevant reported follow-up time points were extracted when available. Where multiple adjusted estimates were reported for the same outcome and time point, the most fully adjusted estimate was prioritized.	These procedures were specified to ensure consistent extraction and clinically meaningful interpretation of the available evidence across heterogeneous studies.
**Endpoint-level evidence-strength appraisal**	A structured endpoint-level classification of evidence strength was not prespecified.	Major outcome domains were qualitatively appraised according to study design, risk of bias, consistency, directness, and breadth and duration of follow-up, and categorized as stronger, moderate, or limited. Formal GRADE was not performed.	This methodological refinement was introduced to improve transparency and clinical interpretability without altering study eligibility, data extraction, or the underlying narrative synthesis.

Abbreviations: CE, Conformité Européenne; FDA, United States Food and Drug Administration; MINORS, Methodological Index for Non-Randomized Studies; RoB 2, revised Cochrane Risk of Bias tool for randomized trials; SAVR, surgical aortic valve replacement; TAVI, transcatheter aortic valve implantation; TAVR, transcatheter aortic valve replacement.

Note: The primary review objective and the principal prespecified clinical, procedural, and anatomical domains were retained. The changes reported above comprise both substantive deviations from the original thesis protocol and methodological refinements introduced during operationalization of the completed systematic review.

Table S4. RoB 2 assessment of randomized-trial-derived studies.

Reference	Study type	RoB 2 overall judgment	Principal reasons for the judgment
van Royen et al. 2025 [39]	Prespecified powered substudy of an open-label randomized noninferiority trial	Some concerns	Open-label trial-derived substudy; possible deviations from intended interventions/crossovers, although endpoint adjudication and imaging assessment were centrally performed.
Feistritzer et al. 2025 [49]	Prespecified 5-year follow-up of an open-label multicentre factorial RCT	Some concerns	Open-label design; incomplete long-term echocardiographic follow-up with a small analyzable subset at 5 years.
Modine et al. 2024 [77]	Post hoc age-subgroup analysis of a randomized clinical trial	Some concerns	Post hoc subgroup analysis; subgroup not separately randomized; increased risk of selective reporting.
Feistritzer et al. 2021 [83]	Open-label multicentre 2 × 2 factorial RCT	Some concerns	Open-label conduct; some risk of bias for softer or assessor-dependent outcomes.

Abbreviations: RCT, Randomized Controlled Trial; PSM, Propensity Score Matching; MC, Multicentre; SC, Single Centre; IPTW, Inverse Probability of Treatment Weighting; CE, European Conformity; ViV, Valve-in-Valve.

Table S5. MINORS assessment of non-randomized studies.

Reference	Design subtype	Overall methodological quality	Principal limitations / dominant bias domains
Worthley et al. 2025 [37]	Prospective single-arm MC international clinical investigation	Moderate	No comparator; device-specific single-arm cohort; limited control of confounding.
Bunc et al. 2025 [38]	Retrospective all-comer registry study with PSM analysis	Higher	Retrospective single-centre registry; residual confounding despite matching remains possible.

Gada et al. 2025 [31]	Postmarket prospective MC nonrandomized single-arm study	Moderate	No comparator; device/procedure-specific cohort; potential selection bias.
Kanehama et al. 2025 [76]	Retrospective observational comparative study (SC)	Moderate	Retrospective single-centre comparative design; short follow-up; limited external validity.
Bihag et al. 2025 [50]	Retrospective observational SC propensity-matched comparative study	Higher	Residual confounding despite matching; single-centre design; valve allocation was not randomized.
Hahn et al. 2025 [57]	Post hoc pooled analysis of trial cohorts	Moderate	Post hoc pooled secondary analysis; selected parent cohorts; selective-reporting risk.
Van Mieghem et al. 2025 [59]	Prospective single-arm MC post-market study	Moderate	No concurrent control group; device-specific post-market cohort.
Kini et al. 2025 [69]	Propensity score-matched registry study (MC)	Higher	Observational nonrandomized comparison; residual confounding despite extensive matching.
Rück et al. 2025 [63]	Retrospective MC registry	Moderate	Retrospective multicentre registry design; no concurrent comparator; short 30-day outcome window.
Nakase et al. 2025 [51]	Retrospective analysis of a prospective registry; 1:1 PSM observational study	Higher	Residual confounding remains possible despite matching; single-country registry-based analysis.
Chen et al. 2024 [54]	Registry analysis / cohort study	Moderate	Observational registry design; no external comparator; outcome-specific analysis.
Frank et al. 2024 [78]	Investigator-initiated MC observational registry study	Moderate	Nonrandomized registry design; temporal and centre-level

			confounding remain possible.
Costa et al. 2024 [58]	Prospective observational registry-based study (SC)	Moderate	Single-centre observational design; modest sample; imaging-based surrogate outcomes.
Mas-Peiro et al. 2024 [79]	MC retrospective analysis of prospectively collected data	Higher	Observational design; residual confounding; discharge-focused echocardiographic assessment.
Elkoumy et al. 2024 [70]	MC retrospective observational study; post hoc registry analysis	Higher	Post hoc registry analysis; residual confounding despite matching/adjustment.
Briedis et al. 2023 [41]	Prospective first-in-human feasibility study; nonrandomized single-arm	Lower	Very small sample; early first-in-human feasibility setting; no comparator.
Zaid et al. 2023 [32]	MC retrospective observational study (limited market release)	Moderate	Retrospective limited-market-release cohort; historical/centre selection bias remains possible.
Chandrasekar et al. 2023 [55]	Retrospective SC observational study	Lower	Single-centre retrospective design; no comparator; short follow-up.
Nikolayevska et al. 2024 [66]	Retrospective SC study	Moderate	Single-centre retrospective design; short-term reporting only; residual confounding.
Reardon et al. 2023 [72]	Prospective multicentre global single-arm study	Moderate	No direct comparator; device-specific single-arm design.
Leone et al. 2023 [52]	Observational retrospective MC registry study with IPTW	Higher	Residual confounding despite weighting; observational registry design.
Sondergaard et al. 2023 [36]	Prospective MC global single-arm investigational study	Moderate	No concurrent comparator; investigational device cohort.

Toggweiler et al. 2022 [80]	Prospective MC registry	Moderate	No comparator; registry design; predominantly short-term follow-up.
Buono et al. 2022 [33]	Retrospective MC registry with PSM comparative analysis	Higher	Residual confounding despite matching; mainly short-term outcomes.
Kim et al. 2022 [81]	Retrospective observational cohort study (SC)	Moderate	Retrospective single-centre cohort; treatment allocation was not random; limited external validity.
Loewenstein et al. 2022 [82]	Retrospective MC propensity-score-matched registry analysis	Higher	Retrospective registry design; residual confounding despite matching.
Deeb et al. 2022 [61]	Propensity-matched cross-cohort analysis of bicuspid and randomized low-risk datasets	Higher	Nonrandomized cross-cohort comparison; residual confounding remains possible despite matching.
Aidietis et al. 2022 [40]	Premarket prospective MC single-arm study	Moderate	No comparator; premarket device experience; moderate sample size.
Voigtländer et al. 2021 [56]	Retrospective MC study	Moderate	Retrospective multicentre design; multivalve heterogeneity; residual confounding.
Van Mieghem et al. 2022 [60]	Prospective MC multinational single-arm observational study	Moderate	No direct comparator; observational post-market single-arm design.
Möllmann et al. 2021 [34]	Prospective single-arm nonrandomized MC study	Moderate	Single-arm nonrandomized study; no control group; device-specific cohort.
Schaefer et al. 2021 [84]	Retrospective SC consecutive cohort	Lower	Small retrospective single-centre cohort; no comparator; short 30-day follow-up.
Modolo et al. 2021 [85]	Prospective SC proof-of-principle feasibility study	Moderate	Single-centre feasibility design; surrogate/imaging-focused endpoints;

			no clinical comparator.
Yamada et al. 2020 [67]	Retrospective single-centre study	Lower	Retrospective single-centre design; predictor-focused analysis; very short laboratory follow-up.
Fontana et al. 2020 [35]	Pooled analysis of two prospective MC nonrandomized single-arm studies	Moderate	Nonrandomized pooled single-arm analysis; no comparator; 30-day outcomes only.
Kaneko et al. 2020 [65]	Retrospective case series	Lower	Extremely small sample; case-series design; no comparator.
Armijo et al. 2020 [53]	Retrospective MC registry cohort	Moderate	Retrospective registry comparison; nonrandomized valve allocation; residual confounding.
Manoharan et al. 2020 [86]	Prospective single-arm MC post-commercialisation study	Moderate	Single-arm post-commercialisation design; no concurrent comparator; short-term endpoint focus.
Chow et al. 2020 [87]	Retrospective cohort study (SC)	Lower	Retrospective single-centre cohort; no direct comparator; modest sample size.
Modolo et al. 2020 [88]	Retrospective MC pooled imaging analysis	Moderate	Retrospective pooled imaging analysis; selected analyzable aortograms; no longitudinal clinical follow-up.
Owais et al. 2020 [68]	Single-centre retrospective comparative study	Lower	Single-centre retrospective design; nonrandomized comparison; modest sample size.
Waksman et al. 2020 [62]	Prospective investigator-initiated MC feasibility trial	Moderate	Feasibility trial with small sample; no randomized valve-platform comparison.

Duran Karaduman et al. 2020 [89]	Retrospective single-centre analysis	Moderate	Retrospective single-centre analysis; descriptive platform comparisons; heterogeneous valve mix.
Tang et al. 2020 [30]	Retrospective MC pilot imaging study	Moderate	Retrospective pilot imaging study; no systematic clinical follow-up; selected imaging endpoints.
Rogers et al. 2020 [29]	Prospective MC CT analysis of low-risk trial participants	Moderate	Selected CT subset of trial participants; single-platform analysis; limited clinical endpoints.
Sengupta et al. 2020 [64]	MC retrospective analysis of prospectively collected data	Moderate	Noncomparative observational design; selected extreme-annulus cohort; moderate sample size.

Abbreviations: CT, Computed Tomography; IPTW, Inverse Probability of Treatment Weighting; MC, Multicentre; PSM, Propensity Score Matching; SC, Single Centre.

Table S6. Representation of valve families/models in the included literature.

Valve family/model	Device category	Variants/generations identified in the included set	Number of included studies explicitly naming this family/model	Nature of representation	Evidence status
Edwards SAPIEN family	BEV	SAPIEN XT; SAPIEN 3; SAPIEN 3 Ultra; SAPIEN 3 Ultra RESILIA	25	Dominant recurrent BEV platform across single-arm, comparative, randomized, registry, imaging, and pooled analyses	Extensive / mature
Medtronic CoreValve/Evolut family	SEV	CoreValve; Evolut; Evolut R; Evolut PRO;	25	Dominant recurrent SEV platform in single-arm,	Extensive / mature

		Evolut PRO+; Evolut FX		comparative, randomized, bicuspid, alignment, registry, and long-term studies	
Boston Scientific ACURATE neo family	SEV	ACURATE neo; ACURATE neo2	12	Recurrent contemporary family in single-arm, within-family generational, post-dilation, platform-comparison, and procedural-strategy studies	Moderate / established
Abbott Navitor	SEV	Navitor; Navitor THV; Navitor TAVR System	4	Mostly dedicated contemporary single-arm/post-market studies, plus one mixed Portico cohort mention	Moderate / early-expanding
Abbott Portico	SEV	Portico; Portico THV with FlexNav delivery system	5	Single-arm, registry, and multi-valve comparative representation	Limited-to-moderate
JenaValve	THV	JenaValve	3	2 alignment/coronary-access papers; 1 CE-mark table	Peripheral
Lotus / Lotus Edge family	Mechanically expandable / legacy THV	Lotus; Lotus Edge	3	Appears only in descriptive small-annulus or	Peripheral / legacy only

				pooled multivalve datasets; no dedicated contemporary evidence stream	
Meril Myval family	BEV	Myval; Myval Octacor	2	Direct comparative/head-to-head representation only	Limited but comparative
Hydra THV	SEV	Hydra THV	2	One dedicated prospective single-arm study plus one mixed SEV cohort mention	Limited
ALLEGRA	SEV	ALLEGRA	1	Single comparative ViV study only	Very limited / isolated
Vienna Aortic Valve Self-Expandable System	SEV	Vienna Aortic Valve Self-Expandable System	1	Single first-in-human feasibility study only	Very limited / early feasibility
Other isolated pooled multivalve platforms	Mixed isolated platforms	Centera (SEV); Direct Flow Medical; INOVARE (BEV)	1 each	Single pooled videodensitometry study	Peripheral only

Abbreviations: BEV, Balloon-Expandable Valve; CE, European Conformity; SEV, Self-Expandable Valve; THV, Transcatheter Heart Valve; ViV, Valve-in-Valve

Table S7. Mapping of the included literature across prespecified domains and factors.

Prespecified domain/factor	Coverage in included studies	Representative valve families/models identified	Type of evidence available	Reporting pattern	Evidence-gap comment
Pacemaker implantation /	Extensive; among the most frequently reported	Evolut R/PRO/PRO+/FX, Portico/Navitor, ACURATE neo/neo2, Hydra, SAPIEN 3/S3	Randomized trials, propensity-matched registries,	Usually discharge/30-day PPI; some 1-	Evidence is PPI-centric;

conduction disturbances	domains, both as a main endpoint and a routine safety outcome.	Ultra/S3 Ultra RESILIA, Myval, Lotus	multicentre/single-centre observational cohorts, post-market single-arm studies, and CT predictor analyses	year dependency or 3-year incidence. Detailed conduction disturbances beyond PPI were inconsistently reported.	standardized conduction endpoints, device-specific mechanistic analyses, and long-term pacemaker dependency data remain limited.
Thrombocytopenia / platelet changes	Very limited; one dedicated study on short-term platelet decline after TAVI.	Sapien XT/Sapien 3; CoreValve/Evolut R/PRO	Single-centre retrospective comparative cohort	Serial platelet counts to nadir within 14 days; focused on short-term platelet drop rather than clinical sequelae.	No multicentre, randomized, or newer-generation platform-specific evidence; platelet biology is underexplored in contemporary THV practice.
Acute kidney injury	Limited; one dedicated AKI comparison and one post-dilation study	Sapien 3; ACURATE neo/neo2	Single-centre retrospective comparative study plus indirect observational	In-hospital/30-day reporting, mainly in contrast-minimization or	Dedicated contemporary AKI data across valve

	reporting no AKI signal.		procedural-strategy evidence	postdilation contexts rather than platform comparisons.	families are sparse and strongly confounded by contrast strategy and baseline renal risk.
Paravalvular leak	Extensive; one of the dominant themes across the included literature.	Navitor, Portico, Evolut family, ACURATE neo/neo2, SAPIEN 3/S3UR, Myval, Hydra, Lotus, Vienna, ALLEGRA	Randomized trials, propensity-matched registries, post-market single-arm studies, core-lab CT/echo analyses, and videodensitometry studies	Usually discharge/30-day mild-or-greater or moderate-or-greater PVL; fewer 1–3-year datasets. Assessment methods were heterogeneous.	Cross-study comparability is limited by variable grading thresholds and core-lab use; long-term clinical significance of residual mild PVL remains incompletely defined.
Mean pressure gradient / hemodynamic performance	Extensive; routinely reported across comparative and single-arm studies.	Navitor, Portico, Evolut family, ACURATE neo/neo2, SAPIEN 3/S3UR, Myval, Hydra, Vienna, ALLEGRA	Randomized trials, pooled/post hoc analyses, propensity-matched registries, ViV cohorts, and first-in-human/CE-mark studies	Discharge/30-day gradients were common; some 1-, 3-, and 5-year follow-up. Supra-annular SEV often showed lower	Newest-generation head-to-head data and long-term durability/bioprosthetic valve

				gradients than BEV.	failure analyses remain limited, especially in special anatomies.
Bicuspid valve anatomy	Moderate but still limited; a few dedicated bicuspid cohorts/comparisons.	Evolut R/PRO, ACURATE neo2, SAPIEN 3	Propensity-matched comparison, multicentre feasibility study, and retrospective registry	Short-term/1-year safety, hemodynamics, PVL, and PPI predominate; phenotype granularity was limited.	No randomized platform comparisons in bicuspid AS and limited stratification by Sievers type, raphe calcification, or aortic morphology.
Horizontal aorta / aortic angulation	Very sparse; only one small case series specifically addressed extremely horizontal aorta.	Evolut R/PRO	Single-centre retrospective case series	Technical feasibility and early outcomes only.	No comparative or larger contemporary studies; generalizability across devices and operators is poor.
Small annulus	Moderate; several dedicated analyses focused on small annuli and	SAPIEN 3, Evolut R/PRO, ACURATE neo, Portico, Lotus	Post hoc pooled trial analysis and multicentre retrospective	Discharge, 30-day, 1-year, and up to 5-year hemodynamic/PPM reporting;	No randomized dedicated trials for small annulus

	PPM/hemodynamics.		comparative registries	clinical differences were less consistent than hemodynamic differences.	with newest devices; limited evidence on newer intra-annular platforms and long-term durability.
Endocarditis	Very limited; reported only as a long-term adverse event in a few broader outcome studies.	Evolut R/PRO/CoreValve	Post hoc randomized trial subgroup and prospective registry follow-up	Incidental event reporting at 3 years, not a prespecified mechanistic domain.	Marked evidence gap: no dedicated platform-level endocarditis studies or standardized comparative adjudication.
Coronary obstruction / coronary access	Limited to moderate; mainly imaging/reaccess studies rather than hard clinical obstruction studies.	Evolut R/PRO/PRO+/FX, ACURATE neo/neo2, SAPIEN 3	Prospective observational/CT studies, pilot imaging analyses, and post-market procedural series	Immediate/30-day angiographic or CT surrogates (cannulation success, overlap, high-risk anatomy) predominated.	Clinical coronary obstruction events were rarely captured; evidence is dominated by surrogate anatomy

					and selected platforms.
Commissural alignment	Moderate; several recent device- and imaging-focused studies.	Evolut FX/PRO+/R/PRO, ACURATE neo/neo2, SAPIEN 3	Prospective post-market studies, observational registry analyses, and CT-based pilot imaging studies	Procedural and CT metrics (alignment, overlap, implant depth, re-access feasibility) were common; long-term clinical correlations were limited.	Few comparative strategy studies and little long-term evidence linking alignment to coronary access, redo TAVR feasibility, or clinical events.
Balloon predilatation / balloon aortic valvuloplasty	Sparse for predilatation; one dedicated balloon-predilatation-omission study, with indirect adjunctive evidence from post-dilation studies.	ACURATE neo2 (predilatation); ACURATE neo/neo2 (postdilation context)	Multicentre retrospective observational study with propensity matching; single-centre observational cohort	Procedural, in-hospital, and predischarge/30-day outcomes; anatomy/calcification influenced strategy selection.	Randomized evidence is lacking, valve-family coverage is narrow, and predilatation versus postdilation questions remain only partially separated.
Cusp calcification	Sparse and mostly indirect.	SAPIEN 3 Ultra; ACURATE neo2 (indirect anatomy/calcification context)	Observational predictor analyses	CT-based calcification was used as a	No dedicated comparative studies

				predictor/covariate for PPI, PVL, or predilatation strategy rather than a stand-alone target.	on cusp-specific calcification patterns by device type and no standardized calcification-based treatment framework.
Left ventricular systolic dysfunction	Very limited; one dedicated comparative study in reduced LVEF.	Sapien XT/Sapien 3/Sapien 3 Ultra vs CoreValve/Evolut R/PRO/PRO Plus	Propensity-matched observational registry study	Discharge, 30-day, 1-year, and 5-year outcomes with emphasis on mortality, gradients, and PVR.	Evidence is confined to a single registry analysis; randomized and newer-platform data in low-LVEF populations are lacking.
Sex differences	Minimal; sex-specific findings were incidental rather than a primary domain.	SAPIEN 3	Post hoc subgroup analysis	Reported as a female subgroup within small-annulus analyses, without dedicated sex-stratified comparative endpoints.	Major evidence gap: almost no dedicated sex-based comparative analyses of platform choice,

anatomy-device interaction, or outcome heterogeneity.

Abbreviations: PPI, Permanent Pacemaker Implantation; AKI, Acute Kidney Injury; CT, Computed Tomography; Echo, Echocardiography; PVL, Paravalvular Leak; CE, European Conformity; AS, Aortic Stenosis; PVR, Paravalvular Regurgitation; LVEF, Left Ventricular Ejection Fraction.

Table S8**.** Identified evidence gaps and reporting limitations.

Evidence gap / reporting limitation	How it appeared in the included literature	Representative domains / examples	Main implication
Concentration on recurrent early endpoints	Reporting focused mainly on pacemaker implantation, paravalvular leak, and early hemodynamic performance, usually at discharge or 30 days.	PPI/conduction outcomes; PVL; mean gradient/hemodynamics	Common early outcomes were well described, but the overall clinical profile remained incomplete.
Sparse coverage of several prespecified domains	Some domains were represented by only one or a few studies and were usually supported by retrospective, exploratory, or post hoc analyses.	Thrombocytopenia; acute kidney injury; endocarditis; horizontal aorta; low LVEF; sex differences	Confidence in these topics was limited and generalizability was reduced.
Limited contemporary head-to-head evidence	Many datasets were single-arm, historical, or nonrandomized comparisons, with fewer direct comparisons between newer-generation platforms.	Navitor; Myval; Hydra; Vienna system; ACURATE neo2; Evolut FX in selected contexts	Relative performance between newer valve platforms remained uncertain.
Short and uneven follow-up	Most studies reported discharge, 30-day, or 1-year outcomes, whereas longer follow-up was inconsistent and available only	Late PVL significance; pacemaker dependency; durability; endocarditis; long-term hemodynamics	Late clinical consequences and durability-related questions remained incompletely characterized.

	in selected datasets.		
Heterogeneous definitions and assessment methods	Grading thresholds, endpoint definitions, and use of imaging core laboratories were not uniform across studies.	PVL grading; conduction disturbances; coronary access/alignment; calcification-based predictors	Cross-study comparison and narrative synthesis were constrained.
Reliance on surrogate imaging or procedural markers	Several domains were studied mainly through CT, angiographic, or procedural feasibility endpoints rather than hard clinical events.	Coronary access/obstruction; commissural alignment; cusp calcification	The clinical relevance of some observed differences remained indirect.
Predominance of observational and single-centre evidence	Outside the most frequently studied endpoints, the evidence base was often retrospective and vulnerable to confounding and selection bias.	Bicuspid anatomy; small annulus; balloon predilatation strategy; AKI; thrombocytopenia	Causal inference and device-specific interpretation remained limited.
Limited phenotype-specific and mechanistic stratification	Subgroup granularity was often insufficient for anatomy-, sex-, or mechanism-specific inference.	Bicuspid phenotype; raphe/calcification pattern; coronary anatomy; sex; low LVEF	Translation to individualized valve selection remained restricted.

Abbreviations: PPI, Permanent Pacemaker Implantation; LVEF, Left Ventricular Ejection Fraction; PVL, Paravalvular Leak; CT, Computed Tomography; AKI, Acute Kidney Injury.

.
